# Design of Polymeric 3D Printable Materials for THz Technology Applications

**DOI:** 10.1021/acsapm.6c01662

**Published:** 2026-06-25

**Authors:** Beatrice Tosetto, Laura Pilozzi, Mauro Missori, Andrea Camellini, Tingwen M. Guo, Candido Fabrizio Pirri, Yannis Laplace, Giancarlo Rizza, Ignazio Roppolo

**Affiliations:** † Department of Applied Science and Technology, 19032 Politecnico di Torino, Corso Duca degli Abruzzi 24, 10129 Turin, Italy; ‡ Department of Chemistry, Biology and Biotechnology, University of Perugia, Via Elce di Sotto 8, 06123 Perugia, Italy; § Center for Sustainable Future Technologies, Italian Institute of Technology, Via Livorno 60, 10144 Turin, Italy; ∥ Institute for Complex Systems, National Research Council (ISC−CNR), Via dei Taurini 19, 00185 Rome, Italy; ⊥ Laboratoire des Solides Irradiés, CEA/DRF/IRAMIS, Institut Polytechnique de Paris, CNRS, 91128 Palaiseau, France

**Keywords:** 3D printing, photonic crystals, terahertz, polymers, photocurable polymers, additive manufacturing, digital light processing

## Abstract

Terahertz (THz) radiation is rapidly gaining attention for applications in biomedical diagnostics, security, and wireless communication. Polymers play a central role in advancing THz technologies, as they are ideal for lenses, filters, waveguides, and metasurfaces. However, material selection is driven by commercial availability rather than by the rational design of their composition, and progress is hindered by the limited availability of polymers that combine low THz losses with high printability. To address this problem, the relationship between polymer composition and THz transparency in photocurable resins is here investigated, establishing practical guidelines for tailoring THz response through resin composition. A comprehensive framework is developed to predict THz properties based on key structural features, including heteroatoms, cyclic structures, secondary forces, and the carbon-to-oxygen (C/O) ratio. In particular, (meth)­acrylic resins with aliphatic backbones exhibit predictable behavior, enabling the estimation of their THz response directly from the C/O atomic ratio. Using the best-performing formulations, photonic crystals for THz modulation were fabricated via digital light processing (DLP) 3D printing. These devices are used to demonstrate how geometric parameters, fabrication precision, and material properties influence the THz devices’ response. Comparisons between THz-optimized formulations and commercial resins highlight the importance of combining high printability and THz transparency in the fabrication of functional devices, demonstrating that proper resin design enables the decrease of the absorption coefficient up to 5 cm^–1^ at 1THz (vs 19–30 cm^–1^ of commercial resins usually employed for 3D-printed THz devices), maintaining a sufficient printability, resulting in an extension of the range of controllable response of the photonic crystals up to 2 THz, well beyond the sub-0.5 THz limit typically reported in the literature for similar structures. This material-driven approach establishes a rational pathway for designing polymers tailored for THz applications while enabling the fabrication of technologically relevant devices with accessible and low-cost 3D printing.

## Graphical abstract

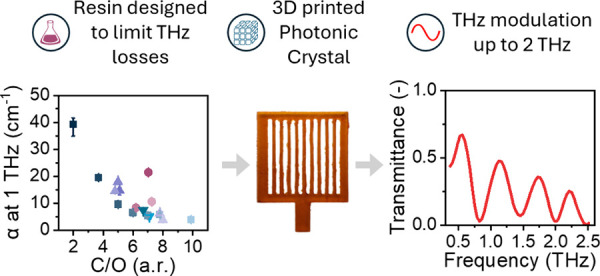

## Introduction

The terahertz (THz) spectral range, spanning between microwaves and the mid-infrared (wavelengths from 30 μm to 3 mm, or frequencies from 0.1 to 10 THz), has long been dubbed as the “last frontier” of the electromagnetic spectrum. Since the 1980s, advances in laser and photonic technologies have transformed this field, driving rapid progress.^1–3^ THz waves combine several remarkable attributes: they are nonionizing and thus biologically safe, are well transmitted through nonpolar materials, and support broadband spectral analysis. These features have spurred technological progress across diverse fields—including biomedical imaging, nondestructive evaluation, quality assurance, security screening, and next-generation wireless communication.^1,3–5^ For instance, THz scanners in security settings outperform traditional metal detectors by identifying materials such as explosives, narcotics, or chemical substances based on their spectral signatures, all while remaining harmless to humans.^4–7^ In the medical field, THz imaging is being explored for the early detection of conditions like skin and breast cancer, due to its sensitivity to the water content of tissues.^3,8–13^ Similarly, THz spectroscopy is increasingly applied for noninvasive quality assessment in industries such as pharmaceuticals, aerospace, and automotive, and for nondestructive diagnostics of cultural heritage materials.^3–5,11–16^ Looking ahead, THz frequencies are expected to play a key role in sixth-generation (6G) wireless systems. Their potential for ultrafast data transmission, low latency, and high capacity makes them ideal for building an interconnected digital environment linking physical, virtual, and human domains. 6G aims to transcend conventional communication, enabling smart manufacturing, real-time digital twins in healthcare, AI-enhanced mobility, and an expansive Internet of Things.^17–20^ Achieving this vision, however, requires networks of objects that are not only connected but also capable of sensing and adapting to their surroundings.

In this context, metamaterials offer a compelling strategy. These engineered structures derive their electromagnetic properties not solely from composition but also from geometry. Through subwavelength architectures, they enable functionalities such as complete absorption, selective reflection, and wavefront shaping across multiple frequency domains, from optics to acoustics.^2,21–23^ Metamaterials can be distinguished into all-dielectric or metal-dielectric (*hybrid)* systems. The choice of materials plays a critical role in the structure’s design, both in terms of the material’s intrinsic properties, which influence the device’s final behavior, and in the effective fabrication feasibility. Hybrid metamaterials typically exhibit higher losses due to stronger material–radiation interactions, whereas all-dielectric metamaterials have been limited by fabrication constraints imposed by conventional subtractive fabrication techniques, which did not allow for reaching the required resolution for a long time.^24–26^

Hybrid metamaterials are usually fabricated with complex procedures derived from semiconductor fabrication, including photolithography, electron-beam lithography (EBL), focused ion beam lithography (FIB), and etching processes, resulting in high costs and limiting scalability.^20,27–29^ However, the development of additive manufacturing techniques, such as Two Photon Polymerization (2PP) and Projection Micro Stereolithography (PμSL), has facilitated the fabrication of THz subwavelength metamaterials, both all-dielectric and hybrid—in the latter case, a subsequent metal deposition step is required.^30,31^

Moreover, the implementation of photonic crystal (PhC) structures for interaction with THz radiation has opened up the employment of more conventional and commercially available 3D printing techniques, such as fused filament fabrication (FFF), digital light processing (DLP), and direct writing (DIW).^32–36^ This is because PhCs are characterized by a periodic modulation of the refractive index on a length scale comparable to the optical wavelength (hundreds of microns for THz-PhCs), thus requiring lower resolution in the production of the metastructures compared to the subwavelength ones.^24,37–39^ However, despite several THz devices being fabricated through 3D printing techniques, the limited availability of transparent 3D printable polymer is a major drawback, leading to limited performance or a narrow THz range of application (up to 0.5 THz for high-resolution printing techniques).^30–36,40^ Notably, to the best of our knowledge, those structures are usually produced using commercially available materials, without focusing on the active design of polymers’ composition, and several studies highlight how the development of 3D printable materials with optimized THz interaction is a key research direction to extend the range of controllable response.^35,40–42^

Despite recent advances, the understanding of how polymeric materials interact with THz radiation remains incomplete—particularly in the case of photopolymers. Existing studies are often fragmented and lack a unified, composition-driven approach. A few investigations have targeted common polymers, highlighting how purely olefinic polymers exhibit higher transparency to THz radiation compared to polymers containing heteroatoms, and this is generally attributed to the presence of polar interactions within the polymer chains that affect THz transmission.^43^ For these reasons, cyclic olefin polymers (COP/COC) are employed as THz waveguides,^44–46^ lens and windows for THz instrumentation,^47^ and as a support layer for integrated devices working in the THz range.^48–50^ However, no comprehensive framework yet exists to describe how polymer composition governs performance in the THz domain.^51–54^ Therefore, filling this knowledge gap is essential, and a deeper comprehension of material–radiation interaction in this range would pave the way for rational design of components tailored to advance THz applications.^55^

To address the current limitations in the understanding of polymer–THz interactions, this study aims to develop a composition-driven framework for designing functional 3D printable materials that can be used to produce devices with programmed interaction with THz radiation. In this work, digital light processing (DLP) is selected as the 3D printing technique due to its low cost, fast production rates of intricate geometries, and higher resolution compared to the other techniques used for THz-PhCs, as FFF and DIW.^41,56,57^ However, DLP employs photocurable resins, which typically are meth­(acrylate) monomers, due to their well-known high reactivity.^23^ However, this implies introduction of heteroatoms, basically oxygen, which appear to reduce the material’s THz transparency, which is necessary for device performance.^41^ Therefore, it was crucial to evaluate the effect on THz absorption of the presence of heteroatoms and other chemical interactions in the structures, in order to define guidelines for the design of 3D printable resins with high THz transparency. To this end, several custom-designed photocurable polymers were developed to span a range of chemical compositions, focusing on key structural features for photocurable formulations such as the presence of heteroatoms (such as oxygen, nitrogen, and sulfur), but also the presence of hydrogen bonding and cyclic moieties. Their THz transmission properties were systematically evaluated to establish relationships between composition and electromagnetic response. Then, all-dielectric THz metagratings were fabricated using three representative resins, spanning a wide range of THz transparency and 3D printability, as a proof of concept of the impact of material selection on THz device performance. The results highlight that rational resin design, combining low THz absorption with high-resolution printability, enables the extension of the controllable response up to 2 THz, well beyond the sub-0.5 THz limit typically reported for similar structures in the literature.^30–36^

Overall, this study lays the foundation for the rational design of photocurable polymers optimized for THz applications and highlights the potential of DLP-based additive manufacturing, combined with the proper design of photocurable formulations, for the development of next-generation THz-active materials and devices.

## Materials and Methods

The photocurable monomers: bisphenol A dimethacrylate (BPADMA), 2-(dimethylamino) ethyl methacrylate (DMAEMA), 1,6-hexanediol diacrylate (HDDA), 2-hydroxyethyl methacrylate (HEMA), isobornyl acrylate (IBOA), *N*-isopropylacrylamide (NIPAM), poly­(ethylene glycol) diacrylate Mn 250 (PEGDA), tricyclo­[5.2.1.0^2,6^] decanedimethanol diacrylate (TCDDMD), 2,2′-thiodiethanethiol (TDET), 1,2,4-trivinylcyclohexane (TVCH), the photoinitiator phenylbis­(2,4,6-trimethyl benzoyl)­phosphine oxide (BAPO), the radical scavenger pentaerythritol tetrakis­(3,5-di*tert*-butyl-4-hydroxyhydro-cinnamate) (PT), and the dyes: *N*,*N*-dimethyl-4,4′-azodianiline (AZO), 2-(2-hydroxy-5-methylphenyl) benzotriazole (BT), and 2-[3-(2H-benzotriazol-2-yl)-4-hydroxy-phenyl]­ethyl methacrylate (BTE) were purchased from Sigma-Merck. The monomers: 1,12-dodecane dioldimethacrylate (DoDDMA), 1,5-hexadiene (HD), octadecyl methacrylate (ODMA), and trimethylol propane triacrylate (TMPTA) were purchased from TCI chemicals, the monomer 1,10-decanedithiol (DDT) was purchased from BLDpharm, while the commercial resin #PlasGray_V2 was provided by Asiga. All chemicals were used without any further purification.

### Polymer Design

Photocurable polymers were designed to obtain specific compositions, choosing the monomers and their percentages. The rationale beyond that (i.e., parameters) is mathematically described by the atomic ratios between some of the present atoms. A total of 18 formulations among (meth)­acrylates and thiol–ene resins were designed, as reported in Table 1.

**Table 1:**
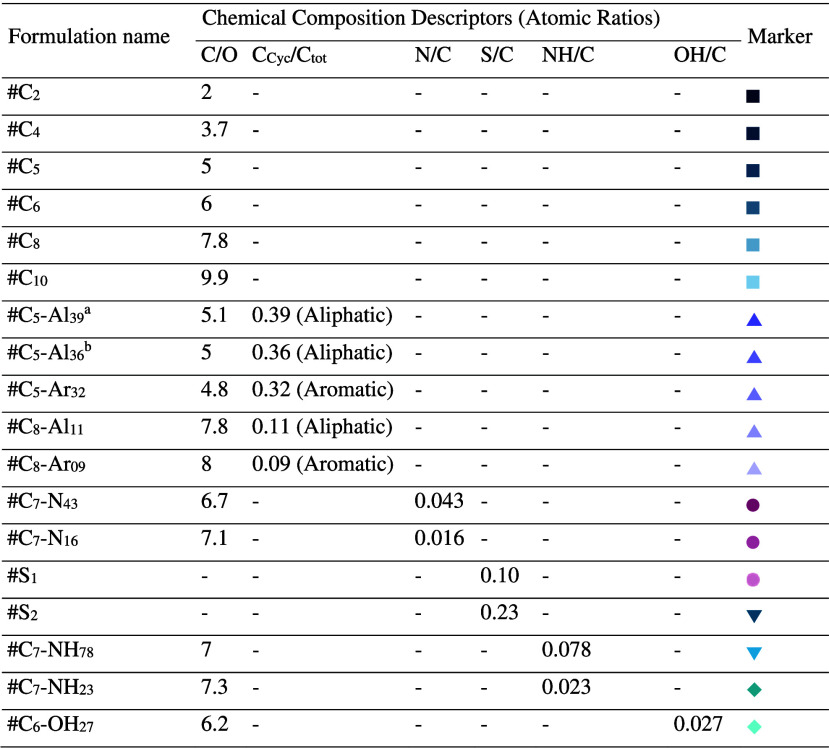
Chemical Composition Parameters of the Designed Formulation^a^

a Thiol–ene resins (#S_1_ and #S_2_) were set on off-stoichiometric ratio, with a + 28.5% ± 1.5% excess of ene.

### Specimens’ Preparation

The designed photocurable formulations were prepared by mixing the monomers as reported in Tables S1 and S2. Formulations were magnetically stirred to be homogenized. Afterward, different specimens were prepared for characterization. Specimens of 1 × 1 cm^2^ areas and different thicknesses were produced by casting using PDMS spacers between two glass microscope slides or Teflon sheet. Irradiation was performed by means of two different photocuring systems: a UV oven equipped with a medium-pressure mercury lamp provided by Asiga (UV-light intensity: 10 mW cm^–2^) and the UV lamp Hamamatsu LC8 (UV-light intensity: 50 mW cm^–2^). The photocuring conditions used for the formulations described in Table S1 are reported in Table S3, while those reported in Table S2 were polymerized in the UV oven for 10 min for each side.

The production of different objects was carried out by using the DLP-3D Printer Asiga MAX X27 UV (build area: 51.8 × 29.2 × 75 mm^3^, light emission at λ = 385 nm, nominal XY-pixel resolution of 27 μm, layer thickness adjustable from 1 to 500 μm). The light intensity, the exposure time, and the layer thickness were optimized for each material and the 3D model to be printed, while for the Asiga commercial resin, Plas Gray V2 was processed according to the printing parameters provided by the manufacturer. The printing parameters are described in the discussion. The building platform was covered with a glass layer to improve the optical quality of the sample’s surface, and after the printing process, two postcuring steps (one per side) were performed for 3 min in a UV oven equipped with a medium-pressure mercury lamp provided by Asiga (UV-light intensity 12 mW cm^–2^) under a nitrogen atmosphere.

### Characterization

The cross-linked fraction (insoluble fraction) and the residual unreacted monomers of the photocured samples were evaluated by means of insoluble fraction tests. The samples were held in a net, weighed, and soaked in chloroform; after 24, h the samples were dried until no further mass variation was detected. The gel content (GC%) was evaluated as reported in eq 1:

$$\mathrm{GC}\%=(\frac{\mathrm{Final}\;\mathrm{weight}}{\mathrm{Initial}\;\mathrm{weight}})\cdot 100$$ 1

Fourier-transform infrared spectroscopy (FT-IR) analyses were performed in attenuated total reflectance (ATR) mode with a Nicolet iS50 FT-IR Spectrometer with diamond crystal (32 scans from 4000 to 500 cm^–1^, resolution of 4.0 cm^–1^). For (meth)­acrylate-based formulations, polymerization was monitored by evaluating—before and after light irradiation—the area below the peak at 810 cm^–1^ (related to C–H bending in the acrylic group), normalized to the one under the peak at 1720–1730 cm^–1^ (C=O bond stretching in the acrylic group);^58^ and the conversion degree (CD) was calculated by eq 2:

$$\mathrm{CD}=\left(1-\frac{A_{\mathrm{polymer}}}{A_{\mathrm{formulation}}}\right)\cdot 100$$ 2

where *A* is the ratio between the underlying areas, evaluated on the formulation spectrum—i.e., before light irradiation, and in the polymers’ spectra—i.e., after light irradiation. Instead, for thiol–ene-based formulations, evaluation was performed both for thiol and vinyl group conversion. The evaluated peaks were at 1630 cm^–1^ for vinyl groups and 2540 cm^–1^ for thiol, and they were compared to the peaks in the range 2760–3020 cm^–1^ (peaks at 2920 and 2850 cm^–1^; related to aliphatic C–H stretching).^58^ FT-IR analyses were conducted on both samples’ sides in duplicate, so the CD results are the average of four measurements (error bars represent the range of data variation).

Light penetration into the liquid formulations during the 3D printing process was established by evaluating the thickness of the polymerized samples obtained by irradiating the resins for different exposure times and with different light intensities (50 mW/cm^2^ for the #C_7_-N_16_ formulation and 59 mW/cm^2^ for the #C_7_ formulation). The thickness was assessed as the average of four measurements (error bars represent the range of data variation), and the data were employed to define the working curves, as defined by Jacobs with eq 3, where the curing depth (*C*_d_—thickness of the cured layer) is expressed as a function of the given energy dose (*E—*product between light intensity and time).^59^ Substituting the dose with *E* = *I* · *t* and simplifying the equation, eq 4 is obtained, and it was used to fit the measured thickness as a function of the exposure time.

$$C_{d}=D_{p}\cdot \ln\left(\frac{E}{E_{c}}\right)$$ 3

$$C_{d}=A\cdot \ln(t)-B$$ 4

Real-time rheological measurements were performed using an Anton Paar rheometer (Physica MCR 302) in parallel-plate mode with the UV lamp Hamamatsu LC8 (UV-light intensity: 50 mW cm^–2^). The gap between the two plates was 0.3 mm; the measurements were conducted at 30 °C under a constant frequency of 1 Hz; and the light was turned on after 60 s to stabilize the system. The variation of storage (*G*′) and loss (*G*″) moduli during irradiation was measured as a function of exposure time. The measurements were carried out in the linear viscoelastic region (LVE) with a strain amplitude of 50%. The LVE was previously evaluated by performing an amplitude sweep measurement at 1 Hz (strain from 0.01 to 500%). The viscosity of the formulations was instead evaluated with a three-step flow curve in which the shear rate varied between 0.001 and 1000 Hz, with a logarithmic ramp. The viscosity was then evaluated as the average value among those characterized by a torque value >0.1 μNm.

The printing fidelity of the 3D-printed object to the original CAD models was evaluated through a 3D optical scanner (E4, 3Shape, sensitivity 4 μm) by comparing the scanned and the reference model. Results are displayed as a colored 3D map of the geometrical deviation from the original CAD, while standard deviations and mean dimensional errors are automatically provided by the software. Multiple structures were analyzed for the designed formulations; the results are given as the average of the data obtained.

The characteristic dimensions of the gratings were evaluated to simulate a more accurate interaction with THz radiation. The measurements were performed with a digital caliper for thickness (average of four measurements) and with image analysis through ImageJ and MATLAB software for the rods’ width (average of 900) and their distance (average of 800 value). Sample images were taken with an optical microscope with a magnification of 2×. These images were then processed with ImageJ software by (i) setting the scale using the reference of 1.00 mm in the picture, (ii) rotating the image to have the metastructure’s rods parallel to the *y* axes, (iii) transforming the image in 8bit and adjusting the contrast (auto mode), (iv) transforming the image in binary format to separate the objects of interest from the background (with the threshold function in auto mode), and (v) cropping the area containing the rods; if necessary a manual correction of the b/w areas was performed. After this process, the black and white values (0–255) of the pixels along 100 horizontal lines (perpendicular to the rods direction) were evaluated in the areas between 15 and 75% of the rods’ length in order to avoid any misinterpretation caused by the object shadows. The obtained data were analyzed with MATLAB, and the rods’ width is obtained by average of 900 values (100 measurements on each of the nine rods) and, for the rods’ distance, by average of 800 values (100 measurements on each of the eight slits—the first and the last one were excluded from the analyses to avoid misinterpretation related to image cropping).

THz time domain spectroscopy was employed to characterize the transmission and the optical properties of polymeric specimens. THz pulses were generated and detected using an ultrafast Ti:sapphire oscillator (80 MHz repetition rate, 100 fs pulse duration, 120 mW average power). A photoconductive antenna (Tera-SED), biased at 10 V and modulated at 10 kHz, generated the THz pulses using 90% of the laser power. The emitted THz radiation was collimated and focused onto the sample with a spot diameter of ∼2 mm, ensuring illumination within the sample area. For detection, the remaining 10% of the laser power served as a gating beam for electro-optic sampling in a 1 mm-thick ZnTe ⟨110⟩ crystal, measuring the transmitted THz pulse. The experimental setup is schematically represented in Figure S1. The time delay between the pump and probe pulses was scanned in two set-ups: over a range of 55 ps in steps of 0.07 ps, resulting in a spectral resolution of ∼19 GHz after Fourier transformation, and over a range of 27 ps—steps of 0.27 ps, resulting in a resolution of ∼37 GHz. Each time-domain waveform was averaged over two scans. Reference scans were acquired without the sample to determine the absolute optical constants. All measurements were conducted in a dry atmosphere to minimize water vapor absorption.

The transmission and the complex refractive index of the metamaterial was extracted from the transmitted THz time traces using established methods.^60^ To compute the transmittance, the time-domain signals were windowed to suppress etalon effects from optical components, Fourier-transformed, and normalized to the reference. Repeated measurements on multiple samples confirmed a measurement uncertainty of <5%. A MATLAB algorithm was developed to determine the thickness that allows to cancel Fabry–Perot oscillation in the optical parameter evaluation, selecting among the measured thickness and 100–200 μm less, with 1 μm increments. Further details are reported in the Supporting Information file on page S-3.

Multiple experiments were conducted for each material, summary data are reported at 1 THz as the average of the measured values, and error bars represent the range of data variation.

### Simulations

The numerical simulations of the metastructures’ THz transmittance were carried out using a one-dimensional Rigorous Coupled-Wave Analysis (RCWA) method, which is widely employed for modeling optical structures with in-plane periodic modulation of the complex refractive index.^61–63^ The method reformulates Maxwell’s equations in the Fourier domain. A total of 81 Fourier harmonics were retained in the field’s expansion; this truncation was selected based on convergence tests of the zeroth order transmission at specific wavelength values under normal incidence. Simulations were performed using the open-source code *rcwa-1d* developed by Pavel Kwiecień distributed under the GNU General Public License version 2 and available at https://sourceforge.net/p/rcwa-1d/news/. The experimental complex refractive index was converted to a wavelength axis. The refractive indices of both superstrate and substrate were set to unity. The grating geometry was defined using both CAD dimensions and the averaged geometrical parameters extracted from the fabricated structures. The incident field was considered at normal incidence with the electric field polarized along the grating rods. For each structure, the calculations returned the wavelength-dependent zeroth-order transmittance and reflectance sampled with a wavelength step of 1 μm. Although multiple diffraction orders are explicitly accounted for in the simulations, only the zeroth-order transmittance is discussed in this work. This choice reflects the experimental configuration, as the limited size and acceptance angle of the THz detector prevent the collection of radiation diffracted into higher orders. In postprocessing, the simulated transmittance spectra were interpolated onto the wavelength grid associated with the material refractive index data. The interpolated spectra were then transformed back from wavelength to frequency, yielding transmittance spectra on a grid with spacing 37.5 GHz.

The modulators were designed as rods gratings of periodicity d and modeled solving Fourier-transformed Maxwell’s equations by series expansion of fields and permittivity.

The structures’ profile was defined through its dielectric function through eq 5, where ε_0_ is the vacuum dielectric function, ε_b_ the dielectric function of the rods’ material, and *F*(*x*) is the sum of products of Heaviside functions, reported in eq 6, in which *L* is the rod width in the *x* direction, $x_{1}=ld$ with l=0,±1,±2,···,±N , is the coordinate of the center of the l th rod, and N is the number of reciprocal lattice vectors (G=2πl/d) included in the expansion, needed for numerical convergence.

$$\varepsilon (x)=\varepsilon_{0}+F(x)(\varepsilon_{b}-\varepsilon_{0})$$ 5

$$F(x)=\lim_{N\to \infty }\;\sum_{l=-N}^{N}\theta [x-(x_{l}-L/2)]\cdot \theta [(x_{l}+L/2)-x]$$ 6

Moreover, given the filling factor of the elementary cell *f*_f_ = *L*/*d*, an average weighted dielectric function was defined by eq 7, which characterizes an equivalent slab model of the grating.

$$\overset{-}{\varepsilon }=f_{f}\cdot \varepsilon_{b}+(1-f_{f})\cdot \varepsilon 0$$ 7

Assuming a harmonic time dependence in the form exp­(iωt), where ω is the frequency of the incident light, and a plane wave propagating in the *xz* plane, the vectorial scattering problem can be separated into two independent scalar problems: (i) one for the electric field parallel to the grooves (s-polarization), (ii) one for the magnetic field parallel to the grooves (p-polarization).

The amplitudes of the partial waves inside the patterned layer and the incoming (incident) and outgoing waves (reflected and transmitted) were defined as 2*N* + 1-dimensional vectors in the Fourier components, following established methods.^64^ So, the electric field components (*i* = *x*, *y*, *z*) in the rods’ structure can then be expressed as eq 8.

$$E_{i}(x,z,\omega )=\Sigma_{m}[A_{m}^{+}\cdot e^{ik_{m}z}+A_{m}^{-}\cdot e^{-ik_{m}z}]\Sigma_{G}E_{i}(G,k_{m})\cdot e^{i(q_{x}+G)x}$$ 8

where *A*_*m*_^+^ and *A*_*m*_^–^ are the amplitudes of forward and backward propagating waves along *z*, *q*_*x*_ is the wave vector component parallel to the surface, *E_i_* (*G*, *k*_*m*_) and *k*_*m*_ are eigenvectors and eigenvalues (solutions to Maxwell’s equations as a generalized eigenvalue problem).

By imposing Maxwell boundary conditions at *z* = 0 and *z* = *L*_*z*_ (perpendicular to the grating), the optical response of the system was described by determining the amplitudes of partial waves and then the reflection and transmission coefficients. This requires solving a system of equations of size 2­(2*N* + 1) × 2­(2*N* + 1):

$$S_{\rho }\cdot \left(\begin{array}{c} A^{+}\\ A^{-} \end{array}\right)=\left(\begin{array}{c} I\\ 0 \end{array}\right)$$ 9

where *S*_ρ_ (with ρ = s, p) are the scattering matrices for s and p-polarization, respectively.^64^

Specifically, solving eq 9 for s-polarization, we obtain

$$r_{G}^{s}=\sum_{m}[A_{m}^{+}+A_{m}^{-}]\cdot E_{y}(G,k_{m})-\delta_{G,0}$$ 10

$$t_{G}^{s}=\sum_{m}[A_{m}^{+}\cdot e^{ik_{m}L_{z}}+A_{m}^{-}\cdot e^{-ik_{m}L_{z}}]\cdot E_{y}(G,k_{m})$$ 11

where *r*_*G*_^*s*^ in eq 10 is the reflectivity and *t*_*G*_^*s*^ in eq 11 is the transmittivity.

## Results and Discussion

### Rationale of the Formulations

The fabrication of 3D-printed all-dielectric THz devices requires the employment of polymers, which should possess a good THz transparency to avoid signal loss, together with high printability, for an accurate models’ production. To date, the limited availability of polymer that combines those characteristics is a major drawback, leading to limited performance or narrow THz range of application (below 0.5 THz), as discussed in the Introduction.^30–36,40^ Therefore, a proper design of the employed polymer is necessary to improve THz device development, and the aim of this work is to define easily implementable guidelines for the rational design of resin composition to achieve targeted THz interactions.

Among the photocurable resins, acrylate and methacrylate are the most commonly used materials in light-induced 3D printing due to their high reactivity.^23^ However, based on the existing literature about polymer response in the THz range, their composition may potentially affect THz transmission. Indeed, purely olefinic polymer shows higher transparency than those containing also heteroatoms, suggesting that polar interactions tend to increase THz absorbance.^1,41,43,51–54^ Consequently, a systematic investigation of (meth)­acrylate optical properties in the THz range was the starting point of this study. Since all the (meth)­acrylic monomers contain at least one ester group, the carbon-to-oxygen atomic ratio (C/O) was set as the key parameter for evaluating the effect of polymer composition on THz transmission.

The first set of formulations was designed to include oxygen as the only heteroatom, mainly associated with the presence of (meth)­acrylic functional groups. It is well-known that bifunctional low-molecular-weight monomers have enhanced printability, since the gel point is reached with low exposure times;^23^ however, based on the considerations discussed above, it is also expected that a high content of (meth)­acrylate functionalities should reduce THz transparency. Therefore, the tested formulations were conceived to find a balance between processability and optical performance. The targeted chemical composition parameters (C/O ratios) are reported in Table 1, and the corresponding monomer mixtures are reported in Table S1; summarizing, formulations with C/O ratios ranging from 2 to 10 were prepared. PEGDA 250 (formulation #C_2_) was included in this set, despite its oxygen content not being exclusively related to ester groups being the monomer of a polyether. This decision has two-fold reasons: on the one hand, the widespread use of PEGDA in 3D printing leads to the desire to include a relevant benchmark material in these analyses; on the other hand, the polyester monomer with C/O ratio = 2 (ethylene diacrylate) cannot be used to produce specimens in pure form, since high rigidity and high shrinkage lead to cracking.

Additional sets of formulations were designed to investigate the effect of other chemical features on the THz transmission. In particular, the influence of cyclic carbon structures (both aromatic and aliphatic), additional heteroatoms (nitrogen and sulfur), and hydrogen bonding was evaluated. To this end, these formulations were defined not only by the C/O ratio but also by additional compositional parameters describing their relevant chemical characteristics. Specifically, the fraction of cyclic carbon was expressed as the atomic ratio *C*_cyc_/*C*_tot_, the presence of heteroatoms was quantified through the N/C and S/C ratios, and the hydrogen-bonding capability was represented by the NH/C and OH/C atomic ratios. The selected formulations are listed in Table 1. Notably, the presence of sulfur as heteroatoms was studied employing thiol–ene resins instead of (meth)­acrylates, allowing us to completely exclude the presence of oxygen.

### THz Transmission Properties

Once the rationale for the formulation design was established, squared specimens were prepared by casting (curing conditions in Table S3), and subsequently they were analyzed using THz time domain spectroscopy (THz-TDS). Transmission THz-TDS analysis measures the electric field of a THz wave passing through a specimen in the time domain (experimental setup in Figure S1); then, the data are converted to the frequency domain via Fourier transform and finally processed to calculate the material’s properties. In particular, the refractive index (*n*) and absorption coefficient (α) were numerically extracted by accounting for the sample thickness in the calculation method.

The set of polymers from #C_2_ to #C_10_ was initially investigated. The impact of the C/O ratio is revealed from the transmittance spectra in the optical range of 0.4–2.5 THz, which is reported in Figure 1a. The graph clearly shows that THz signal transmission increases with the C/O atomic ratio in the polymer matrix.

**Figure 1:**
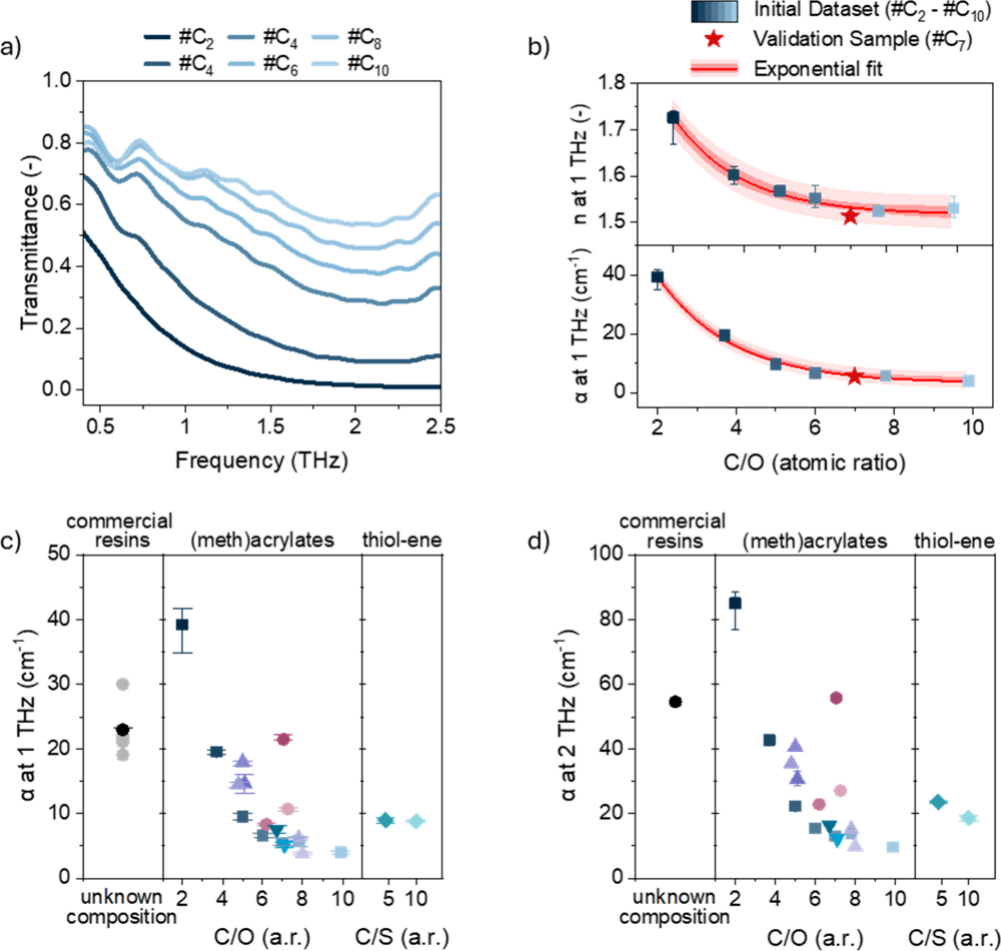
(a) THz transmittance of 0.5 mm thick samples with different C/O ratios and any other studied chemical features; (b) exponential decay fits of the absorption coefficient and refractive index (including 95% confidence and prediction bands), derived from polymers #C_2_, #C_4_, #C_5_, #C_6_, #C_8_, and #C_10_ and validated with the additional composition #C_7_; (c,d) summary of the absorption coefficient at 1THz (c) and 2THz (d) of the studied polymers. Marker color and shape correspond to the formulations reported in Table 1. Commercial resins are included for comparison (black: #PlasGray_V2, gray: literature data from ref 41).

The optical parameters n and α were then evaluated, in order to extract the material’s properties of those polymers, and all the spectra are reported in the Supporting Information (Figure S2). After that, to further explore their correlation with the C/O ratio, the values at 1 THz were compared across those formulations, and they are reported in Figure 1b as squares colored with a blue gradient; darkening in the color indicates increasing oxygen content. These values were then fitted in function of the carbon content (C/O ratio), revealing an exponential decay (red curves in Figure 1b). Both fits are of good quality with an *R*-square of 0.990 for α and 0.967 for *n*, and all the fitting parameters are reported in Table S4. To validate the observed trend, an additional formulation having a different C/O ratio was used to prepare specimens to be tested in the THz range. Consequently, formulation #C_7_ (50%_wt_ of ODMA + 50%_wt_ of DoDDMA, C/O ratio = 7) was designed, and the absorption coefficient and refractive index of the polymerized samples at 1 THz are reported in Figure 1b with red stars, falling within the 95% confidence band for α and the 95% prediction band for *n*. For completeness, the full optical parameters’ spectra are reported in Figure S3. This confirms the insights obtained from the tested data set, giving a useful tool for the prediction of materials properties in the THz range and demonstrating that, despite its simplifying assumptions, the mode based on the C/O ratio remains effective and enables rapid prediction of resin behavior in the THz range.

The exponential decay trends of the optical parameters with the C/O ratio are similar for both *n* and α, although the variation is significantly more pronounced for the latter (90%), while the refractive index exhibits a more modest 10% reduction—highlighting the much greater sensitivity of THz transmittance to changes in absorption coefficient than to variations in the refractive index. Specifically, the refractive index drops from 1.73 to 1.54, and the absorption coefficient decreases markedly from 40 to 4 as the C/O ratio rises from 2 to 10. For this reason, the following discussions will mainly focus on the absorption coefficient, whereas the corresponding results for the refractive index will be provided in the Supporting Information.

Extending the analyses to the monomers’ backbone chemical composition, the optical parameters of all the tested material were evaluated (Figures S4–S8), and the values at 1 and 2 THz are summarized in Figure 1c,d for α and Figure S9a for *n* in function of the carbon content, in order to observe the chemical composition effect also at higher frequencies. In both figures, marker color and shape correspond to the those reported in Table 1, where the marker shape indicates the characteristic chemical composition descriptors and darkening of the color represents increasing series (blue squares: only oxygen as heteroatoms, violet triangles: cyclic structures, sky-blue inverse triangles: nitrogen as heteroatoms, cyan diamonds: sulfur as heteroatoms, and pink circles: presence of hydrogen bonds).

Analyzing the different chemical features targeted, cyclic structures (violet triangles) showed an impact on the absorption coefficient only at high content and without a clear trend (full spectra in Figures S6 and S7). Indeed, samples with high carbon content but a low amount of cyclic structures—such as #C_8_-Ar_09_ and #C_8_-Al_11_—exhibit optical parameters similar to the reference formulation, e.g., without any cyclic components (#C_8_). In contrast, when the content of cyclic structures increases—as in #C_5_-Al_39_^a^, #C_5_-Al_36_^b^, and #C_5_-Ar_32_—the absorption coefficient increases if compared to #C_5_. On the other hand, the refractive index does not exhibit a consistent trend across the three analyzed formulations: while #C_5_-Al_36_^b^ and #C_5_-Ar_32_ show higher values compared to the reference material (#C_5_), #C_5_-Al_39_^a^ displays a nearly identical refractive index (Figure S6).

This lack of a clear and consistent trend suggests that other factors beyond the presence of cyclic moieties may contribute to the observed optical response. In particular, since all the tested materials were obtained by means of photopolymerization, the extent of conversion may play a relevant role. In fact, during the polymerization reaction, carbon double bonds are converted to single bonds and this conversion is usually incomplete, depending on parameters such as chain mobility and viscosity.^65,66^ Consequently, to not oversight the observation reported and to assess whether the presence of unreacted groups might influence the interaction of the material with THz radiation, the polymerization efficiency was examined. Gel content measurements and conversion degree results (Table S3—full FT-IR spectra: Figures S10–S13) show a high gel content (>94%) for all the formulations, confirming the formation of well-cross-linked polymer networks. However, when focusing on the conversion degree (CD), some samples exhibit values significantly below 90% (70% ± 4% for #C_5_-Al_35_^b^, 47% ± 16% for #C_5_-Ar_35_, and 73% ± 4% for #C_8_-Ar_10_). This suggests that the distinct behavior in the refractive index observed in the samples containing ring structures may be more closely related to differences in double-bond conversion rather than the mere presence of cyclic moieties. The detailed investigation of this aspect is out of the scope of this paper; however, future planned activities aim at better determining this aspect also in light of recent publications focused on the relation between THz absorption and irradiation dose.^67^

Concerning the presence of heteroatoms other than oxygen, they do not appear to significantly affect the THz optical response, at least within the investigated compositional range. In particular, polymers containing nitrogen in the form of tertiary amines (#C_7_-N_43_ and #C_7_-N_16_—sky-blue inverse triangles) exhibit only limited variations in their optical parameters (full spectra in Figure S5), with values close to those of the reference polymer with the same C/O ratio but without nitrogen (#C_7_).

A similar behavior is observed in thiol–ene polymers (cyan diamonds), where the absorption coefficient is not affected by the presence of S atoms, i.e., α is independent of the C/S ratio (#S_1_: C/S = 10 | #S_2_: C/S = 4.3). On the other hand, a significant variation in the refractive index is obtained. To assess whether the observed behavior is related to differences in double-bond conversion, both gel content and thiol/vinyl conversion were evaluated (Table S3—full FT-IR spectra: Figure S12). Thiol conversion was high (>95%) in all samples, while the vinyl conversion was different than the expected, which was ∼80% due to the ene excess in the thiol–ene stoichiometry. Specifically, #S_1_ samples showed a higher vinyl conversion (84.6% ± 4.0%), suggesting the occurrence of partial homopolymerization of carbon–carbon double bonds, reaching a nearly complete gel content (∼100%). In contrast, #S_2_ exhibited a lower vinyl conversion (62.6% ± 3.2%) and gel content (60%), due to a much slower polymerization as the reaction is not completed even after 40 min of irradiation. These differences in polymerization process further support the hypothesis that residual unsaturated bonds play a key role in determining the refractive index, as already observed in polymers containing cyclic structures. Nevertheless, the possible relationship between residual double-bond concentration and THz optical properties remains to be clarified and requires further investigation.

Finally, the increasing presence of polar −OH and −NH groups (#C_7_-NH_78_, #C_7_-NH_23_, and #C_6_-OH_27_—pink dots) results in a strong increase of both optical parameters (full spectra in Figure S4). This behavior can be reasonably related to the formation of hydrogen bonds within the polymer structures. Indeed, systems containing nitrogen by lacking hydrogen-bonding capability (e.g., tertiary amines: #C_7_-N_43_ and #C_7_-N_16_—sky-blue inverse triangles) show only minor variations in their optical parameters (full spectra in Figure S5).

The optical properties in the THz range of the designed polymers were compared with those of commercial resins commonly employed in DLP 3D printing (absorption coefficient: Figure 1c,d and refractive index: Figure S9a). Literature data, reported as gray dots, were taken from ref 41, where commercial formulations, selected for their suitability in THz applications in terms of printing resolution and processability, were characterized up to 1.4 THz. In addition, the commercial resin #PlasGray_V2 was experimentally characterized in this work to extend the measured interaction range up to 2.5 THz and is reported in the figures as black dots (full spectra in Figure S9b).

As shown in Figure 1c,d, commercial resins exhibit high absorption coefficients (19–30 cm^–1^ at 1 THz and 55 cm^–1^ at 2 THz), with values comparable to those of polymers characterized by a low C/O ratio or by the presence of hydrogen bonds in their structure. It should be noted that the exact composition of these commercial resins is undisclosed, and they are generally described as mixtures of (meth)­acrylate monomers and oligomers. Nevertheless, #PlasGray_V2 is known to contain monomers with a high concentration of oxygen-bearing groups as well as −NH moieties. Such chemical features are consistent with the observed high absorption, which ultimately limits the effective use of these materials to relatively low-frequency THz applications, where signal attenuation remains acceptable.^41^

Summarizing the data collected, two factors emerged as particularly important in the design of photocurable formulations to obtain high THz transparency: a high C/O ratio and the absence of intermolecular hydrogen bonds. Additionally, high conversion seems crucial to minimize residual double bonds, which contribute to an increased refractive index. On the basis of these results, the formulations that exhibited the best THz transparency were #C_7_, #C_8_, #C_10_, #C_8_–Al_11_, #C_8_-Ar_09_, and #C_7_-N_16_, which show absorption coefficients in the range 5–6 cm^–1^ at 1 THz and 9–15 cm^–1^ at 2 THz, so, in principle, these can be good candidates for 3D printing testing, with better performance than reported in the literature.^40^

### Optimization of the 3D Printing Process

Once those guidelines for the rational design of resin composition to achieve targeted THz interactions were defined, it was possible to work on the resins’ 3D printability, in order to achieve a correct reproduction of the THz devices. Among the studied resins, formulations #C_8_, #C_10_, #C_8_-Al_11_, and #C_8_-N_43_ were excluded for their low content of bifunctional monomers (ranging from 2 to 15%), which in preliminary testing compromised printability by limiting control over reaction propagation. Additionally, #C_8_-Ar_09_ was excluded because the presence of BPADMA gives crystallization issues to the formulation (melting point around 70 °C), making the 3D printing process more challenging, since it may require heating of the formulation, while it is intended to perform fabrication at room temperature.

As a result, among the transparent formulations, only #C_7_ and #C_7_-N_16_ met all criteria and were selected for further 3D printing tests. Unfortunately, none of the two provide satisfactory printing fidelity—that is, the ability to accurately reproduce the details of the Computer-Aided Design (CAD) file, as shown in Figure 2a. This is probably due to the high penetration of light in the formulations, which is highlighted in the working curves reported in Figure 2b, which results in uncontrolled polymerization both in the *z* direction and the *x*–*y* plane. Therefore, the introduction of additives to enhance 3D printing resolution was deemed essential. Commonly used additives to improve 3D printing are radical scavengers and dyes, the first primarily influence the control of polymerization in the *x*–*y* printing plane, whereas the seconds mainly regulate the curing depth.^23,68^ To this end, the effect of three different dyes (*N*,*N*-dimethyl-4,4′-azodianiline—AZO, 2-(2-hydroxy-5-methylphenyl) benzotriazole—BT, 2-[3-(2H-benzotriazol-2-yl)-4-hydroxy-phenyl]­ethyl methacrylate—BTE) and one radical scavenger (pentaerythritol tetrakis­(3,5-di*tert*-butyl-4-hydroxyhydro-cinnamate—PT) was systematically investigated, with the dual objective of improving print fidelity and assessing their impact on the material’s interaction with THz radiation.

**Figure 2:**
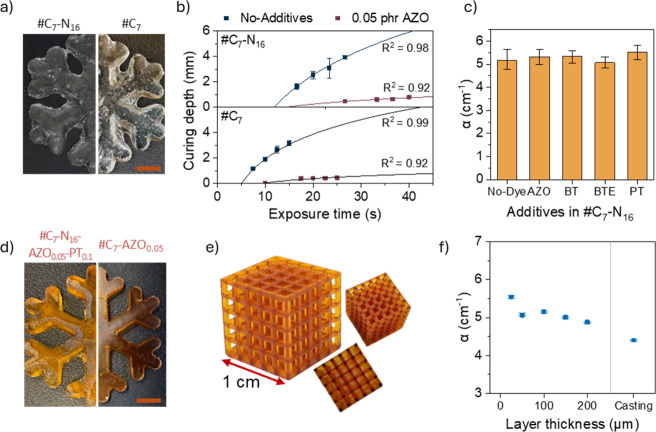
(a) Comparison of 3D-printed snowflakes with #C_7_-N_16_ vs #C_7_ (scale bar of 0.5 cm); (b) light penetration variation in #C_7_-N_16_ and #C_7_ formulations before and after adding 0.05 phr of AZO; (c) α variation of #C_7_-N_16_-based formulations with different additives; (d) comparison of 3D-printed snowflakes with #C_7_-N_16_-AZO_0.05_-PT_0.1_ vs #C_7_-AZO_0.05_ (scale bar of 0.5 cm); (e) 3D-printed complex structure with formulation #C_7_-AZO_0.05_, (f) summary of the absorption coefficient at 1 THz of #C_7_-N_16_-AZO_0.05_-PT_0.1_ samples obtained by 3D printing with different layer thicknesses compared to casting—all samples were 1 mm thick.

As a first step, their effect on the optical parameters was evaluated employing #C_7_-N_16_-based formulations and adding the different additives (formulations composition in Table S2). Importantly, these additives had only a minimal and negligible effect on the absorption coefficient at 1 and 2 THz (Figures 2c and S14a), preserving the material’s transparency. So, it was possible to study their efficacy in improving the printing fidelity.

For #C_7_-N_16_, the addition of the AZO dye significantly reduced the curing depth, as confirmed by the working curve in Figure 2b, outperforming other dyes (BT and BTE—Figure S14b) even at lower concentrations. Moreover, due to the high content of monofunctional monomers, the radical scavenger was also added as it helps in controlling the polymerization in the lateral *x*–*y* plane, leading to more precise geometries after irradiation.^23^ Based on these considerations, formulation #C_7_–N_16_ was optimized by adding 0.05 phr (per hundreds resin) of AZO dye and 0.1 phr of the radical scavenger (PT), obtaining the formulation #C_7_-N_16_-AZO_0.05_-PT_0.1_; composition is provided in Table S5. In contrast, for #C_7_, only 0.05 phr of AZO dye was employed to improve the printing resolution, without adding the radical scavenger since it has a higher content of bifunctional monomer, which inherently limits lateral polymerization and thus allows better control of the propagation of the curing reaction in the *x*–*y* plane. In this case, the optimized formulation is named #C_7_-AZO_0.05_; composition is provided in Table S5.

Before employing these resins for 3D printing, their rheology and photoreactivity were evaluated. The viscosity was assessed as 2.8 ± 1.4 cP for #C_7_-N_16_-AZO_0.05_-PT_0.1_, and 4.5 ± 1.6 cP for #C_7_-AZO_0.05_ (Figure S15a), in good agreement with DLP viscosity requirements;^69^ and the formulation reactivity was studied by means of photorheological analysis (results in Figure S15b). From the comparison of the photorheology of the formulations with and without the additives, it is confirmed that the additives do not affect the reactivity of the systems, as the dye only affects the curing depth and the radical scavenger, the not-irradiated regions. Instead, comparing the two optimized formulations, a marked difference in the reactivity is present: #C_7_-N_16_-AZO_0.05_-PT_0.1_ has an induction time of 15 s, and the gel point is reached after 18 s, while #C_7_-AZO_0.05_ starts to polymerize after 5s of irradiation, and the gel point is obtained after only 8 s. This difference is mainly related to their intrinsic different ratios between the monofunctional and bifunctional monomers, and it is also reflected on the parameters optimized for the printing process, which are reported in Table S6, and finally on the printing fidelity obtained with the two optimized formulations (Figure 2d). In fact, although in both cases the fidelity to the original CAD is strongly improved by the additives, #C_7_-AZO_0.05_ exhibited the best performance, allowing the successful printing of complex three-dimensional shapes (Figure 2e).

Finally, to assess whether the layer-by-layer nature of the printing process could compromise the material’s optical performance, THz-TDS analyses were carried out on 3D-printed #C_7_-N_16_-AZO_0.05_-PT_0.1_ specimens. Therefore, the transmission properties of samples with identical total thickness (1 mm) but varying printing layer thicknesses (from 25 to 200 μm—printing parameters in Table S7) were tested, and the values at 1 THz are reported in Figures 2f and S16a, while the full spectra are provided in Figure S16b. The results showed a slight increase in absorption when the layer thickness decreases—likely due to scattering at layer interfaces—while the refractive index remained nearly unchanged compared to cast specimens.

The interpretation of these data is not trivial, and deeper analyses are required. However, since an effect on the interaction with THz is present, even if limited, an accurate selection of the layer thickness is required in order to not affect THz transmission.

### 3D-Printed Photonic Crystals: Impact of Material Selection

Once the correlation between polymer composition and THz interaction was understood and being able to 3D print transparent formulations, it was possible to produce photonic crystals in order to study their efficacy in the THz modulation and to evaluate the impact of the materials’ properties.

The first set of tests performed aims at evaluating how the intrinsic THz optical properties of the materials and their 3D printability influence the overall THz response of the devices. To this end, we compare the two THz-optimized formulations—#C_7_-AZO_0.05_ and #C_7_–N_16_-AZO_0.05_-PT_0.1_—which differ in their printability—with a commercial resin, #PlasGray_V2, which offers good printing fidelity but poor transparency in the THz range.

The photonic crystal selected for this study is a square grid labeled as PhC_0.25, which is composed of a periodic arrangement of parallel rods 0.25 mm wide, spaced 0.75 mm apart, and 0.45 ± 0.05 mm thick (Figure 3a). In particular, two different structures with the same periodic arrangement, but having a different number of rods, were 3D printed. The first one is 6 mm × 9 mm (patterned area: 4 mm × 4 mm), while the second one is 12 mm × 16 mm (patterned area: 9 mm × 9 mm); since the size of the THz beam used in the measurements ranges from 1 to 2 mm in diameter, it is small enough to selectively probe the central region of the metastructure, ensuring that the data primarily reflect the intrinsic optical response of the patterned area, minimizing contributions from edge effects or possible imperfections at the structure boundaries, and so minimizing a difference between the two structures.

**Figure 3:**
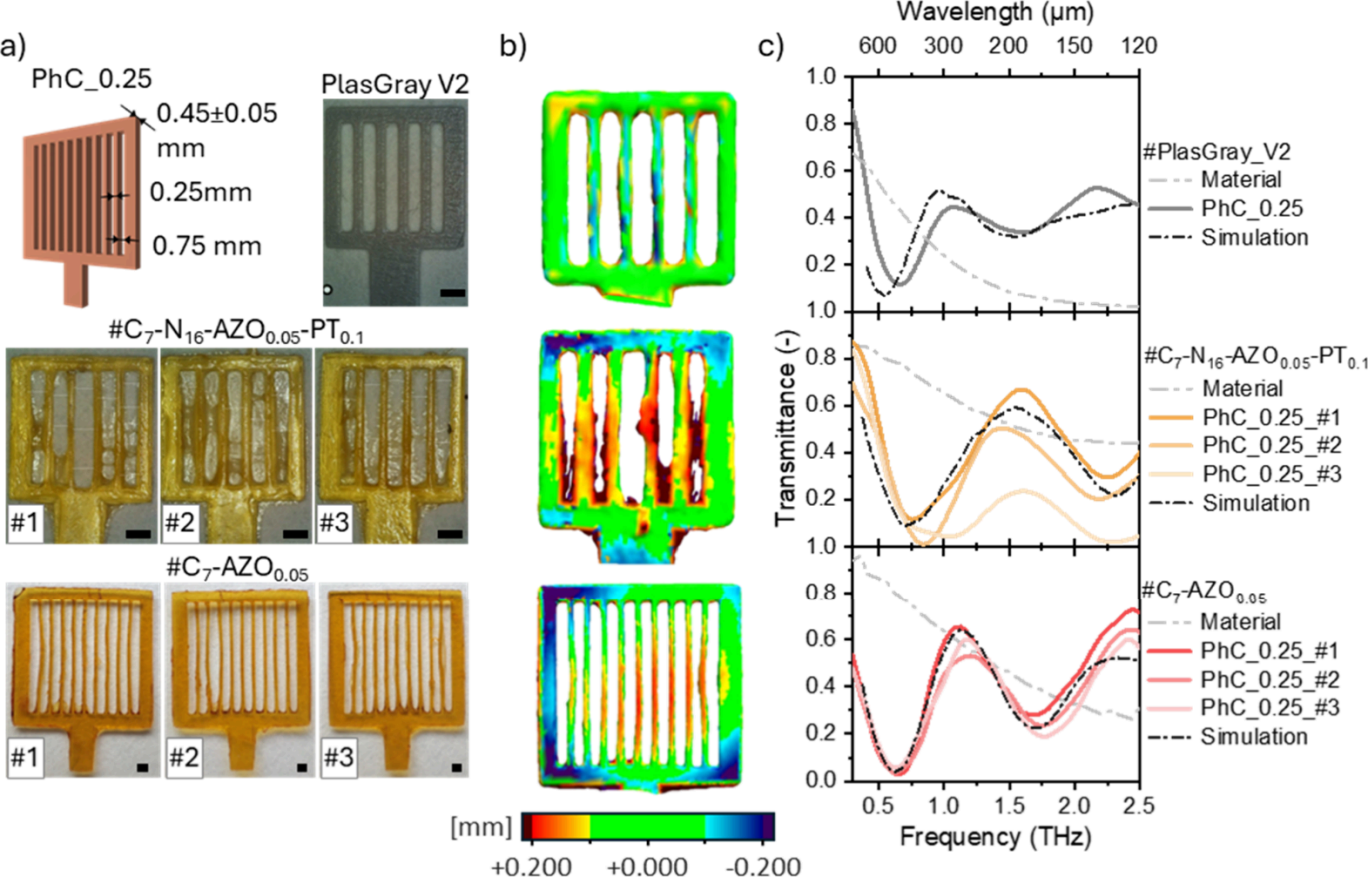
(a) PhC_0.25 CAD and 3D-printed specimens obtained with formulations #PlasGray_V2, #C_7_-N_16_-AZO_0.05_-PT_0.1_, and #C_7_-AZO_0.05_ (scale bar of 1 mm); (b) printing fidelity obtained with formulations #PlasGray_V2, #C_7_-N_16_-AZO_0.05_-PT_0.1_, and #C_7_-AZO_0.05_ (top to down), i.e., the difference between the original cad file and the 3D-printed construct shown as a colormap; (c) THz transmittance spectra of PhC_0.25 metastructures printed with #PlasGray_V2, #C_7_–N_16_-AZO_0.05_-PT_0.1_, and #C_7_-AZO_0.05_, compared with the casted materials (gray dashed-dotted lines) and RCWA simulations (black dashed-dotted lines).

PhC_0.25 metastructures were 3D printed with each resin (Figure 3a—parameter in Table S6), and printing fidelity was assessed via 3D scanning (Figures 3b and S17–S19). Finally, their THz transmission was evaluated, and the transmittance spectra are reported in Figure 3c, along with the transmittance of fully solid casted reference samples of identical thickness (dashed-dotted lines).

In detail, #PlasGray_V2 exhibited good printability, allowing the fabrication of objects with highly defined geometry and uniform rod cross sections (Figures 3b and S17), with a low standard deviation (0.06 mm) and a mean dimensional error of only 0.02 mm, resulting in a satisfactory match between the measured transmittance and the expected one. However, because of its low intrinsic THz transparency, the material strongly absorbs radiation, limiting the impact of the photonic crystal on the transmitted signal. As a result, the THz transmittance remains nearly flat above 1 THz, showing a minimal modulation.

The simulations performed on #C_7_-N_16_-AZO_0.05_-PT_0.1_ structures indicated that photonic crystals produced with that resin were expected to have a more defined modulation of the THz transmission compared to #PlasGray_V2, due to the higher transparency of the material itself. The main features expected were a pronounced valley at 0.71 ± 0.04 THz (9% transmittance vs 76% for the bulk material) and an enhanced transmittance at 1.58 ± 0.04 THz (59% vs 50). However, despite printing optimization, the specimens showed clear defects (Figures 3b and S18), such as variations in rod geometry and poor uniformity along the length of the rods, which led to significant deviations from the original CAD, with a standard deviation spanning from 0.14 up to 0.87 mm and mean errors ranging from 0.03 mm to 0.08 mm. This poor printability of the material caused an inconsistent THz modulation among the different specimens. This correlation is further confirmed by the closer agreement of PhC_0.25_#1 and #2 specimens with the simulated spectrum, as they have better printing fidelity than PhC_0.25_#3 (St. dev. of 0.14 and 0.16 mm vs 0.87 mm).

Finally, photonic crystals printed with #C_7_-AZO_0.05_ showed higher printing fidelity than #C_7_-N_16_-AZO_0.05_-PT_0.1_, allowing the fabrication of devices with regular and homogeneous rods’ dimension and spacing (Figures 3b and S19), with a lower standard deviation (0.14–0.24 mm) and a lower mean dimensional error (0.02 ± 0.01 mm—very similar to that obtained with #PlasGray_V2). This good printability enabled excellent consistency of the response of the different specimens and good agreement with the simulated values, while the high transparency allowed to achieve high THz modulation (from 63 to 3% of transmittance).

This comparison clearly illustrates the pivotal role of material selection to extend the controllable interaction range of THz devices, highlighting the need to balance the THz transparency and printing fidelity when selecting materials for functional THz structures. While the commercial resin #PlasGray_V2 provides excellent print precision, its poor optical properties limit its usefulness in THz applications. Conversely, C_7_-N_16_-AZO_0.05_-PT_0.1_ is highly transparent but suffers from reproducibility issues due to limited printability. Formulation no. C_7_-AZO_0.05_ offers the best compromise between these two aspects, enabling the fabrication of well-defined and repeatable photonic crystals with effective and controllable THz transmission.

### 3D-Printed Photonic Crystals: Impact of Structure Geometry

Once a resin that allowed to combine the desired properties of printability and THz absorbance was found for the development of THz devices, the influence of the structures’ geometry on the THz modulation was investigated. In addition to the refractive index contrast between the rods and the surrounding medium—which can enhance both diffraction efficiency and spectral selectivity—the structural parameters of the patterned design also play a significant role in shaping its optical response.^64,70^

In particular, the grating period governs the onset of propagating diffraction orders, with larger periods allowing a greater number of diffraction orders. The width of the rods, in combination with the period, defines the filling factor—i.e., the portion of the grating period occupied by the rods—thereby modulating the effective refractive index of the structure. Both period and width directly impact the phase-matching conditions for coupling between guided modes and free-space radiation and thus influence the resonance strength and coupling efficiency. Furthermore, the rod thickness controls vertical optical confinement, affecting the formation of leaky guided modes and Fabry–Pérot-like resonances due to multiple internal reflections. These dependencies become evident through comparative analysis of structures with systematically varied geometrical parameters.

To this end, the optical responses of two periodic array designs both 3D printed using the #C_7_-AZO_0.05_ formulation were compared: PhC_0.25 (Figure 3a) already defined in the previous paragraph, and PhC_0.4 (Figure 4a), which is a 1.0 mm-thick grid with rods 0.4 mm wide and spaced 0.6 mm apart.

**Figure 4:**
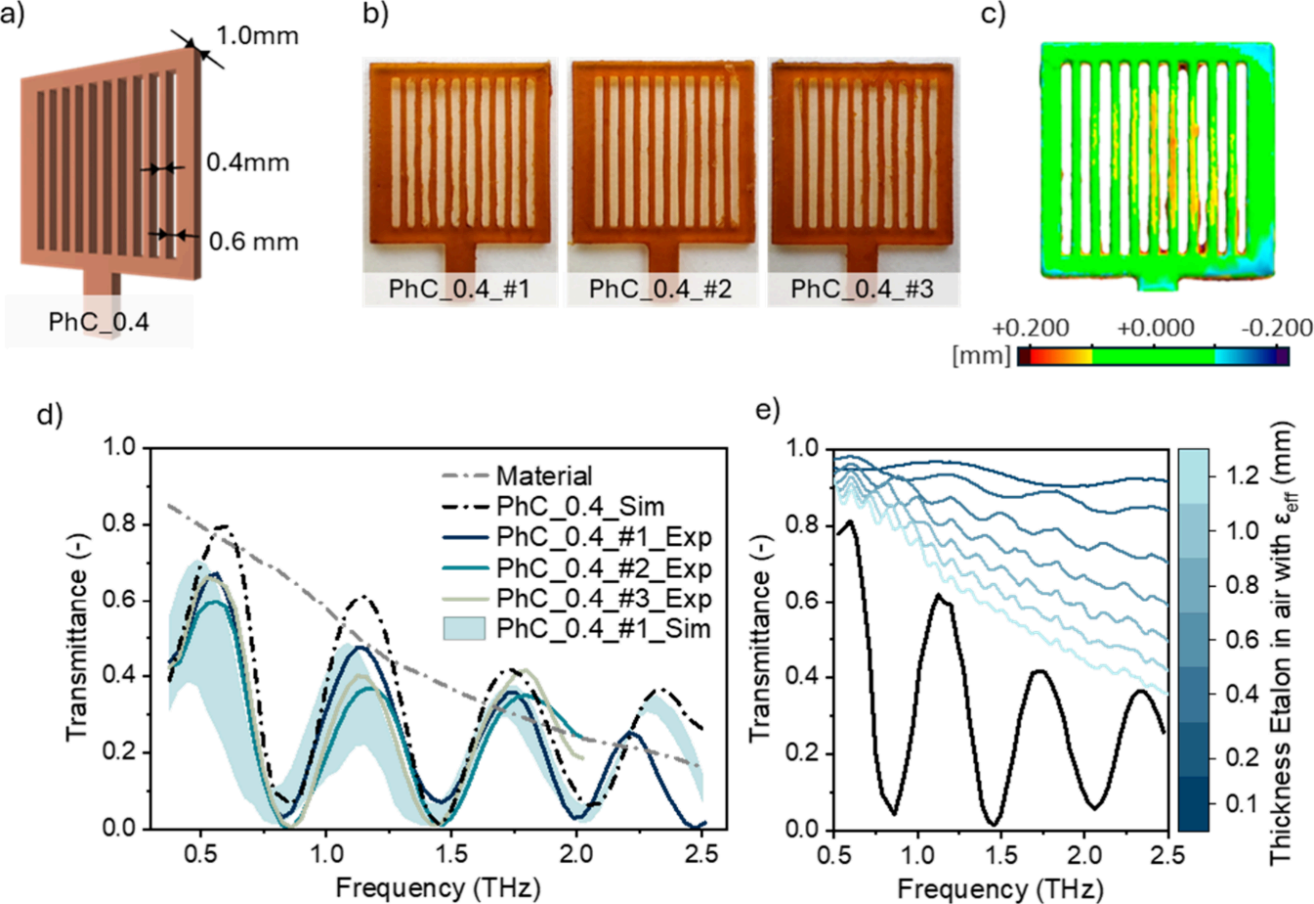
(a) PhC_0.4 CAD model, rods’ width: 0.4 mm, rods’ distance: 0.6 mm, thickness: 1 mm; (b) pictures of the 3D-printed specimens with formulation #C_7_-AZO_0.05_; (c) printing fidelity heat map obtained comparing 3D-scanned model of PhC_0.4_#1 and the original CAD model, on the bottom three cross sections are reported, comparing the CAD model (orange) and the 3D-scanned model (blue); (d) THz transmittance modulation obtained with the photonic crystals compared to the material response (1 mm-thick sample) and the expected transmittance obtained through simulations (dash-dot black spectrum: simulation with CAD metastructure dimension | light blue band: simulation with PhC_0.4_#1 measured dimensions); and (e) additional simulations of the PhC_0.4 structure transmittance (black lines), compared with the transmission of a homogeneous etalon model (colored lines) with a fill-factor-averaged dielectric function and varying thickness.

Three PhC_0.4 models were 3D printed (samples #1, #2, #3), and they are shown in Figure 4b. Their printing parameters are listed in Table S6, while their THz transmittance response is reported in Figure 4d. Compared to the bulk material, in this case also, the structure effectively modulates THz transmission, with photonic bandgaps at 0.86 ± 0.04 THz (98.8% ± 1.2% absorption) and 1.42 ± 0.04 THz (96.6% ± 2.6% absorption). Moreover, the THz modulation is consistent among the three specimens and closely matches the expected behavior of the designed grid (black dash-dotted curve in Figure 4d), particularly in the alignment of the maxima and minima of transmission oscillations. This is because the printing fidelity reveals excellent accuracy with a mean value of 0.02 ± 0.01 mm and a standard deviation of 0.05 ± 0.01 mm, resulting in an in-tolerance region (error <0.1 mm) of 72.0% ± 2.8%. Additionally, cross-sectional comparisons confirm high geometric fidelity (Figure 4c and extended in Figure S20).

However, some discrepancies in transmission intensity are observed between the experimental and theoretical data. To clarify this, the real dimensions of the PhC_0.4_#1-printed sample were measured (rod width: 0.506 ± 0.018 mm | rod distance: 0.509 ± 0.046 mm) and used as input for additional simulations, allowing the definition of a transmission envelope that accounts for the geometric deviations introduced during the 3D printing process.

By comparing the experimental data with this transmittance envelope, it becomes clear that the variations in rods’ spacing and width primarily affect the transmission intensity. This adjustment enables an excellent agreement with the measured modulation amplitude, particularly below 2.0 THz, thereby validating both the simulation methodology and the fabrication process. Nevertheless, a final calibration of the modulation response based on the actual device dimensions appears necessary for precise control in practical applications, in order to mitigate fabrication imperfections.

Finally, comparing the characteristic wavelength involved in the THz modulation of PhC_0.25 (Figure 3c) and PhC_0.4 (Figure 4d) printed with formulation #C_7_-AZO_0.05_, PhC_0.4 confirmed to have a narrower modulation envelope compared to PhC_0.25, as expected from the design of the structures, and this is due to the increased thickness, which enhances the oscillation of the transmission due to multiple internal reflections. To validate this, additional modeling was performed. These analyses allow to propose a conservative interpretation of the physical mechanism underlying the observed spectral response of PhC_0.4. The comparison with the homogeneous etalon shows that a simple effective-medium description, based on a fill-factor-averaged dielectric function ε_eff_, can reproduce only a smooth transmission background but fails to account for the large modulation depth and deep minima observed in the RCWA-calculated grating response (Figure 4e). Therefore, the oscillations seen in the transmittance spectra of the realized structures (Figure 4c) are typical of finite periodic slabs and come from interference effects and modal coupling. Periodicity generates diffraction orders, while thickness acts like a slab giving rise to multiple internal reflections. Indeed, when light enters the grating region, part reflects at the top interface, part propagates inside, then reflects at the bottom interface. As wavelength changes, the phase-matching condition for waves bouncing back and forth inside the finite slab is periodically satisfied, giving rise to an oscillatory transmittance. In a uniform slab, only smooth sinusoidal fringes appear, while Iin periodic structured slabs, multiple modes propagate with different propagation constants, so creating multimode beating, and not just simple Fabry–Perot fringes. Finally, the phenomenological fit (Figure S21 red line) of the zeroth-order transmittance (black dots) shows that the oscillatory transmission has a periodicity of 0.59 THz and that is superimposed on a smoothly decaying envelope resembling the transmission of an effective etalon with comparable thickness: its presence is consistent with a frequency-dependent loss contribution that progressively damps the transmission maxima.

Those results confirm the role of the devices’ architecture on the control of the interaction with the radiation, confirming that it is possible to obtain a fine control of the THz transmission employing fast and economic technique as DLP 3D printing and demonstrating that a systematic design of the photocurable formulation is crucial to obtain efficient structures, allowing to extend the range of controllable interaction up to 2.0 THz.

## Conclusions

As THz radiation continues to gain importance across diverse application fields, the understanding of matter–radiation interactions becomes essential, providing the foundation for designing and fabricating functional devices. In this work, clear and practical guidelines to synthesize THz transparent photocurable resins are established, systematically correlating the molecular features of custom-synthesized photocurable resins with their THz transmission properties. The investigations performed here revealed that the THz properties of (meth)­acrylic resins with purely aliphatic backbones can be quantitatively predicted as a function of the carbon-to-oxygen atomic ratio of the polymer structure, and two key factors were identified as crucial for achieving high THz transparency: a high carbon-to-oxygen atomic ratio, and the absence of intermolecular hydrogen bonding, being able to reach absorption coefficient values in the ranges 5–6 cm^–1^ at 1THz and 9–15 cm^–1^ at 2 THz. Despite the simplifications inherent in the proposed approach, these guidelines provide a straightforward framework for the rational design of low-loss THz materials, which can be readily implemented without the need for advanced synthetic capabilities or specialized instrumentation.

Leveraging this knowledge, three resins were compared for the fabrication of efficient THz modulators using vat polymerization digital light processing (DLP) 3D printing: two THz-optimized formulations (#C_7_-AZO_0.05_ and #C_7_-N_16_-AZO_0.05_-PT_0.1_) and a commercial resin (#PlasGray_V2), taken as representative of the state of the art in 3D-printed THz devices. The results highlight the need to combine high printability with THz transparency in order to achieve functional devices and demonstrate that this balance can be effectively attained through proper design of the photocurable formulations, allowing to reach an absorption coefficient values around 5 cm^–1^ at 1 THz and 12 cm^–1^ at 2 THz for #C_7_-AZO_0.05_ and #C_7_-N_16_-AZO_0.05_-PT_0.1_ polymers, which is significantly lower than the response of commercial 3D printable resins commonly employed for THz devices (19–30 cm^–1^ at 1 THz and 55 cm^–1^ at 2 THz).

Formulation #C_7_-AZO_0.05_ results as the best-performing resin among the analyzed in terms of transparency and 3D printability. Photonic crystals produced with this formulation achieved good printing fidelity (mean value: 0.02 ± 0.01 mm; St. Dev.: 0.05 ± 0.01 mm), enabling fine modulation of THz transmittance, with strong attenuation at 0.86 ± 0.04 THz (98.8 ± 1.2% absorption) and 1.42 ± 0.04 THz (96.6 ± 2.6% absorption), as expected from simulations. This composition-driven approach provides a rational pathway for designing polymers optimized for THz applications, ensuring that both material properties and manufacturability are addressed, and this enables extending the range of applications up to 2.0 THz for DLP 3D-printed THz devices, well beyond the sub-0.5 THz limit typically reported for similar structures in the literature.

Beyond the specific systems studied, this integrated strategy opens avenues for the scalable development of application-specific terahertz materials and devices. Future work will extend this framework to a broader range of chemistries and structural architectures, advancing toward 4D printing. Additionally, this study represents the first step toward the fabrication of metamaterials and metasurfaces, which can be employed to enhance the performance of devices and their potential applications. Finally, the use of low-cost vat 3D printing enables distributed on-demand fabrication of next-generation THz devices with versatile and multifunctional capabilities, fostering wider adoption and democratization of THz technology.

## Supplementary Material



## Notes

Polymers compositions and their full THz-spectra; THz-TDS setup; and data analysis and the optimization of the 3D printing process (PDF)
