# Supplementary material for: Design of Polymeric 3D Printable Materials for THz Technology Applications
Source: ACS Appl Polym Mater. 2026 Jun 25;8(13):10702–15. doi: 10.1021/acsapm.6c01662 (PMC13366651; doi:10.1021/acsapm.6c01662)
Supplement: Supplementary file 1 [file ap6c01662_si_001.pdf]

Supporting information

Design of polymeric 3D printable materials for THz technology applications

Beatrice Tosetto[1,2,3], Laura Pilozzi[4], Mauro Missori[4], Andrea Camellini[1], Tingwen M. Guo[5], Candido Fabrizio Pirri[1,3], Yannis Laplace[5], Giancarlo Rizza[5], Ignazio Roppolo[1,3*]

[1]Department of Applied Science and Technology, Politecnico di Torino, Corso Duca degli Abruzzi 24, 10129, Turin, Italy,

[2]Department of Chemistry, Biology and Biotechnology, University of Perugia, Via Elce di Sotto 8, 06123, Perugia, Italy,

[3]Center for Sustainable Future Technologies, Italian Institute of Technology, Via Livorno 60, 10144 Turin, Italy,

[4]Institute for Complex Systems, National Research Council (ISC-CNR), Via dei Taurini 19, 00185 Rome, Italy

[5]Laboratoire des Solides Irradiés, CEA/DRF/IRAMIS, Institut Polytechnique de Paris, CNRS, 91128, Palaiseau France

*Email :ignazio.roppolo@polito.it

Polymer design

Table S 1. Designed formulation composition

Formulation[1]	Monomers	Composition (Percent by Weight)	Formulation[1]	Monomers	Composition (Percent by Weight)
#C_2	PEGDA	100.0%	#C_5-Al_{39}[a]	IBOA<br>PEGDA<br>DoDDMA	70.3%<br>12.1%<br>17.6%
#C_4	HDDA<br>ODMA	78.0%<br>22.0%	#C_5-Al_{36}[b]	TCDDMD<br>ODMA<br>DoDDMA	66.0%<br>11.0%<br>23.0%
#C_5	DoDDMA	100.0%	#C_5-Ar_{32}	HDDA<br>BPADMA<br>DoDDMA	21.9%<br>58.3%<br>19.8%
#C_6	DoDDMA<br>ODMA	71.0%<br>29.0%	#C_8-Al_{11}	TCDDMD<br>ODMA<br>DoDDMA	21.0%<br>69.0%<br>10.0%
#C_8	TMPTA<br>ODMA	15.0%<br>85.0%	#C_8-Ar_{09}	ODMA<br>BPADMA<br>DoDDMA	63.6%<br>18.2%<br>18.2%
#C_{10}	TMPTA<br>ODMA	4.0%<br>96.0%	#C_7-N_{43}	DMAEMA<br>DoDDMA<br>ODMA	41.0%<br>2.0%<br>58.0%
#C_7-NH_{78}	PEGDA<br>NIPAM<br>ODMA	4.7%<br>51.2%<br>44.1%	#C_7-N_{16}	DMAEMA<br>DoDDMA<br>ODMA	16.0%<br>27.0%<br>58.0%
#C_7-NH_{23}	PEGDA<br>NIPAM<br>ODMA	11.6%<br>15.9%<br>72.5%	#S_1	DDT<br>TVCH	60.0%<br>40.0%
#C_6-OH_{27}	DoDDMA<br>HEMA<br>ODMA	5.3%<br>21.1%<br>73.7%	#S_2	TDET<br>TVCH<br>HD	56.0%<br>23.0%<br>21.0%
[1]to all formulation 0.5 phr of BAPO are added					

Table S 2. Formulation composition for the evaluation of the additive effect on the THz transmission.

Formulation name	Additive[1]		Monomers (wt%)
	phr	name	
#C_7-N_{16}-$AZO_{0.05}$	0.05	AZO	DMAEMA 16%<br>DoDDMA 27%<br>ODMA 58%
#C_7-N_{16}-$BT_{0.5}$	0.5	BT	
#C_7-N_{16}-$BTE_{0.5}$	0.5	BTE	
#C_7-N_{16}-$PT_{0.1}$	0.1	PT	
[1]to all formulation 0.5 phr of BAPO are added			

Table S 3. Casting photocuring conditions and polymeric samples Conversion Degree and Gel content.

	Polymerization process	Conversion Degree	Gel content
#C_2	2 min | UV oven	91%±3%	100%
#C_4	1 min | UV oven	90%±1%	98%
#C_5	3 min | UV oven	88%±4%	100%
#C_6	4 min | UV oven	94%±2%	99%
#C_7	4 min | UV oven	82±2%	-
#C_8	4 min | UV oven | 40°C	96%±1%	98%
#C_{10}	10 min | UV oven | 40°C	97%±3%	90%
#C_7-NH_{78}	4 min | UV lamp | 60°C	94%±3%	90%
#C_7-NH_{23}	20 min | UV oven + 30 min | 65°C	97%±3%	97%
#C_6-OH_{27}	10 min | UV oven | Teflon	98%±0%	94%
#C_5-Al_{39}[a]	1 min | UV oven	95%±2%	100%
#C_5-Al_{36}[b]	1 min | UV oven	70%±4%	100%
#C_5-Ar_{32}	6-10 s| UV lamp | 60°C	47%±16%	94%
#C_8-Al_{11}	10 min | UV oven | 40°C	98%±1%	98%
#C_8-Ar_{09}	6-10 s| UV lamp | 60°C	73%±4%	96%
#C_7-N_{43}	85 min UV oven	96%±2%	73%
#C_7-N_{16}	10 min | UV oven | overnight 65 °C	91%±3%	100%
#S_1	30 min | UV lamp	Th: 94.9%±3.9%<br>Vin: 84.6%±4.0%	100%
#S_2	40 min | UV lamp	Th: 96.4%±3.3%<br>Vin: 62.6%±3.2%	60%

THz-TDS set-up and Optimal thickens algorithm

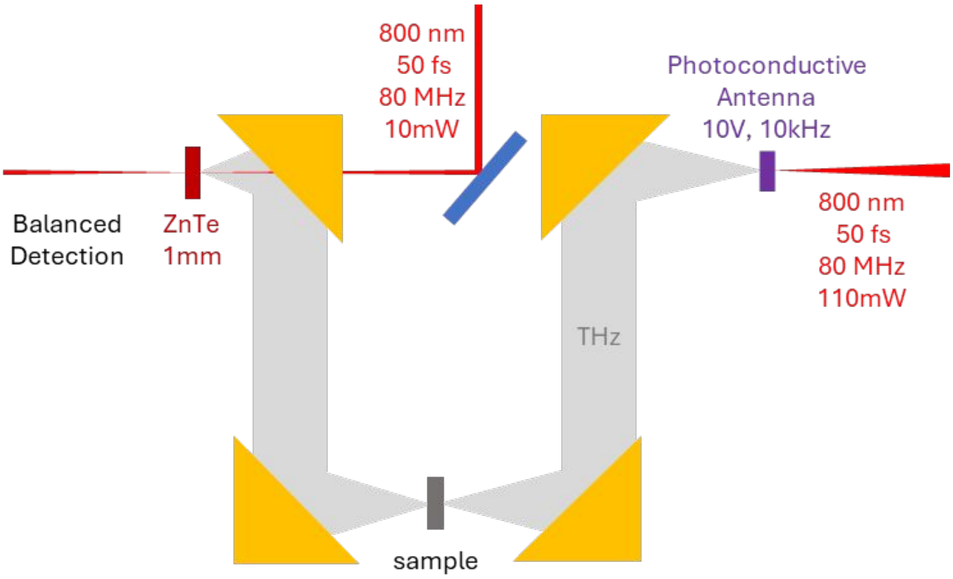

Figure S 1. Schematic representation of the THz-TDS experimental set-up

THz time-domain spectroscopy (THz-TDS – setup in Fig. S1) collects data in the time domain, which is converted to the frequency domain using Fourier transformation. Optical parameters, such as refractive index (n) and absorption coefficient (α), can be numerically extracted considering the sample's

thickness in the calculation method. But to achieve a satisfactory cancellation of Fabry-Perot oscillation in the calculated optical parameters, the necessary precision in the thickness value (0.001 mm) is higher than the one obtained by a micrometer screw gauge (0.02mm).[1–3]

Typically, thickness optimization is done manually by observing the oscillations at various thicknesses. To handle large datasets and establish a systematic approach, a MATLAB algorithm was developed to determine the optimal thickness that minimizes these oscillations. Optical parameters were evaluated for different thickness values, usually from the measured thickness to 100-200μm less, with 1 μm increments. Then, an oscillation index was defined for each thickness as the mean value of the variance between the oscillating curve and a target one, obtained by smoothing the first. This process is repeated both for the refractive index and the absorption coefficient and the optimal thickness is selected as the one that allows to minimize the refractive index oscillation. An ~~acceptable~~ error range is then evaluated by considering the thickness that allows to variate both *n* and *α* oscillation index below some threshold values empirically determined (usually +5%, but a +10% was employed if an error range lower than 10μm was previously evaluated). This acceptable range was employed to account for potential errors and approximations in the method.

THz Optical parameters

From Figs. S2-S8 the optical parameters in the THz range of the polymeric samples whose polymerization is described in Tab. S3 is shown. In all cases, a good superimposition of the optical parameters evaluated for the different thicknesses is obtained in the range 0.4-2.0 THz, confirming the possibility to generalize the discussion to the radiation interaction with the materials instead of that with the samples. Notably, some variations are registered in the range between 2.0 and 2.5 THz, in which a thickness dependency may be present, showing that generally thinner samples have a maximum in the absorbance around 2.0 THz, while thicker samples have it a bit shifted towards higher frequencies (2.2-2.5 THz).

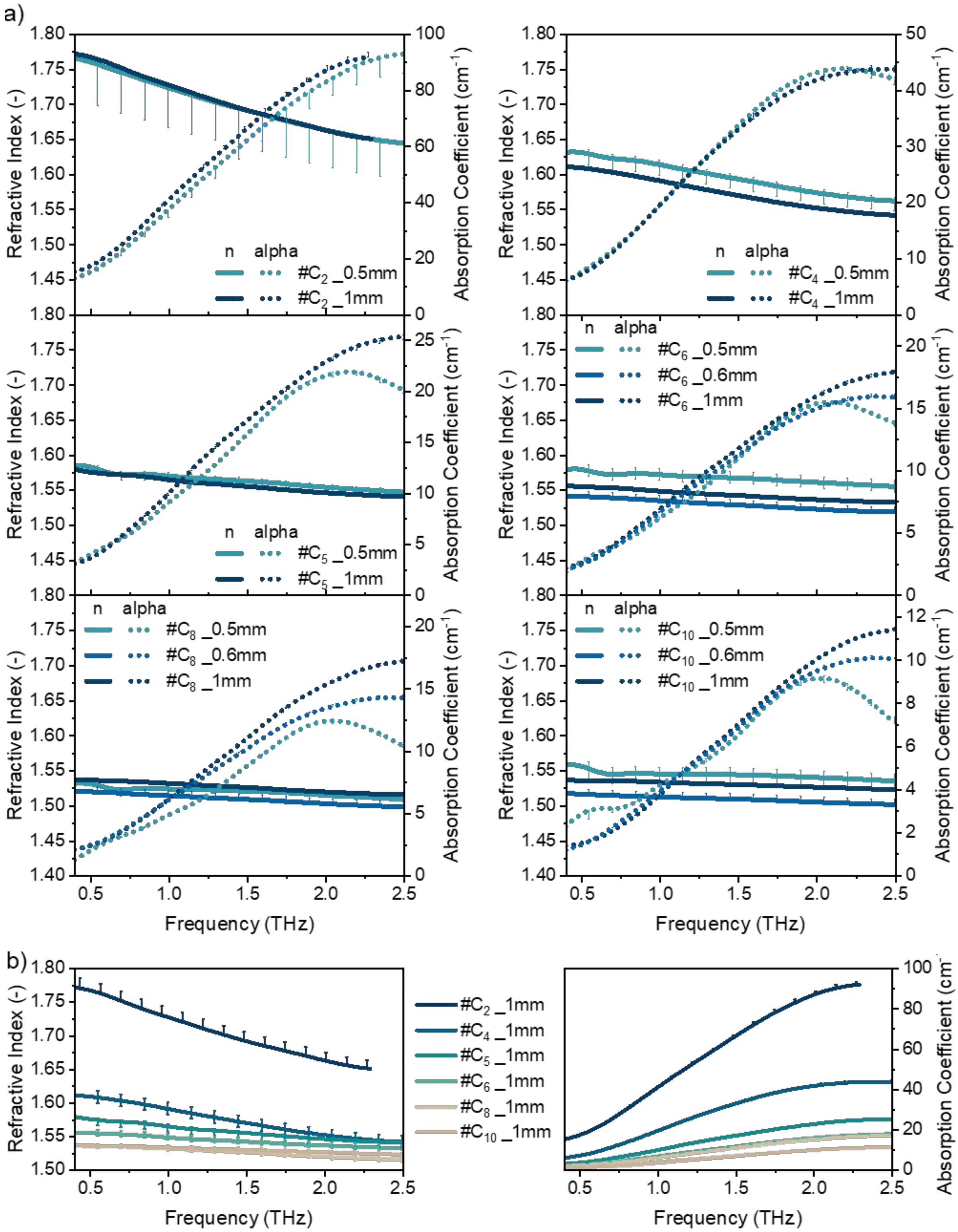

Figure S 2. Absorption coefficient and refractive index for formulations #C_2 to #C_{10}: (a) Spectra obtained from samples with varying thicknesses; (b) Comparative analysis of the optical parameters across different formulations, using samples 1mm-thick.

*Table S 4. Fitting parameters for n and α dependency from the carbon content (C/O ratio) evaluated for material #C_2, #C_4, #C_5, #C_6, #C_8, and #C_{10} following the equation y = y0 + A*exp(R0*x)*

	n at 1THz	α at 1THz
y0	1.52314 ± 0.00732	3.28047 ± 0.70377
A	0.57921± 0.08225	104.79709 ± 8.15041
R0	-0.52573 ± 0.07278	-0.53124 ± 0.03981
R-Square	0.96752	0.99025

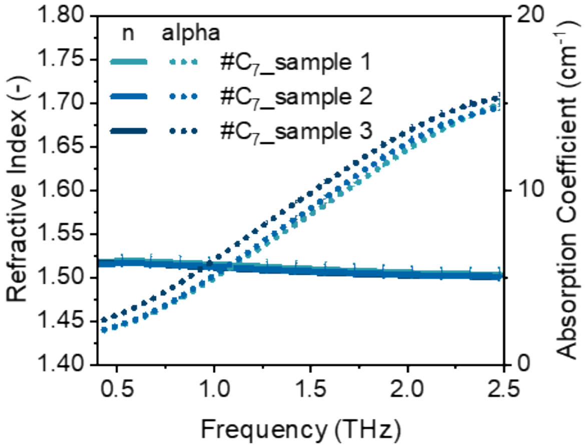

Figure S 3. Absorption coefficient and refractive index for #C_7 obtained from 3 samples 1mm-thick.

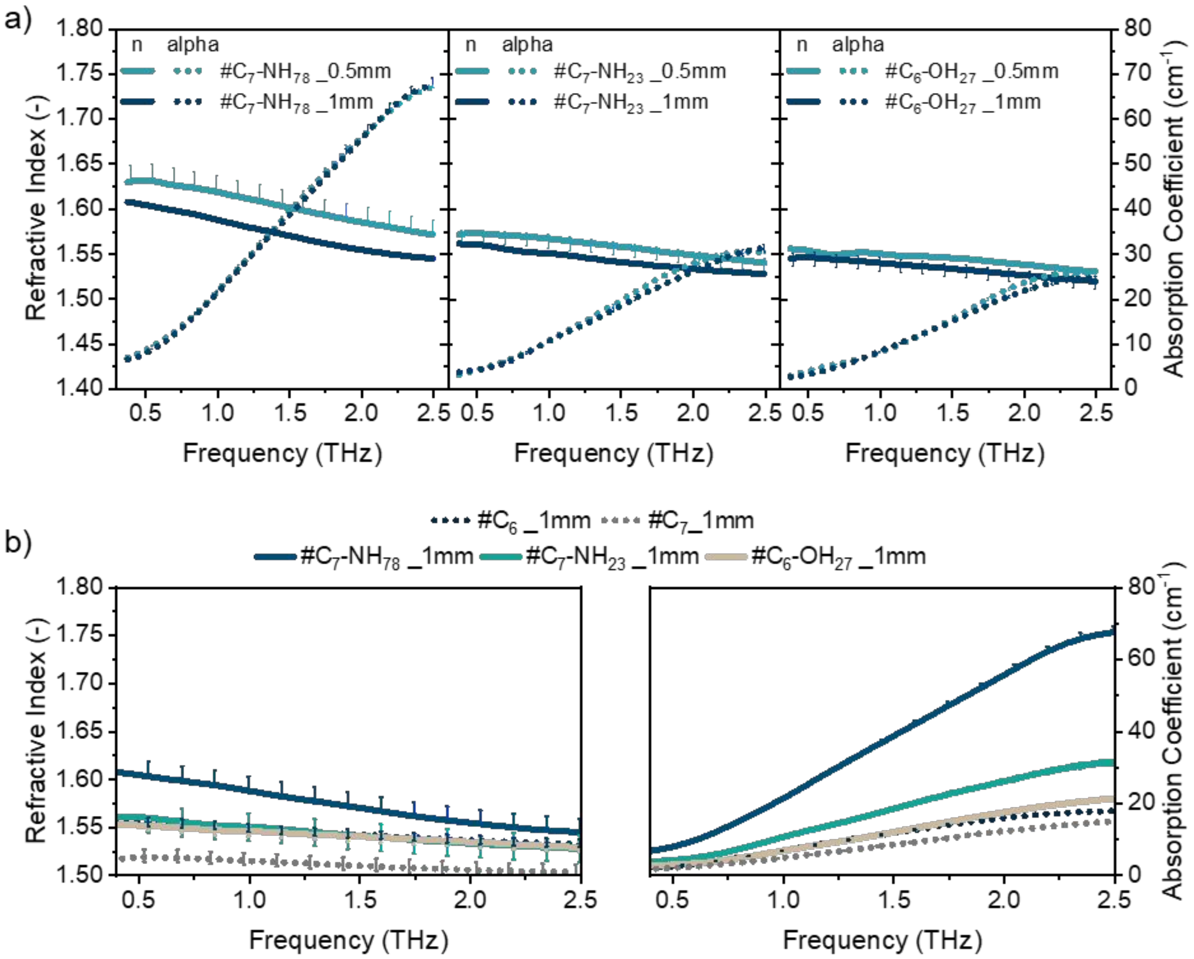

Figure S 4. Absorption coefficient and refractive index for formulations #C_7-NH_{78}, #C_7-NH_{23}, #C_6-OH_{27}: (a) Spectra obtained from samples with varying thicknesses; (b) Comparative analysis of the optical parameters across different formulations, using samples 1mm-thick.

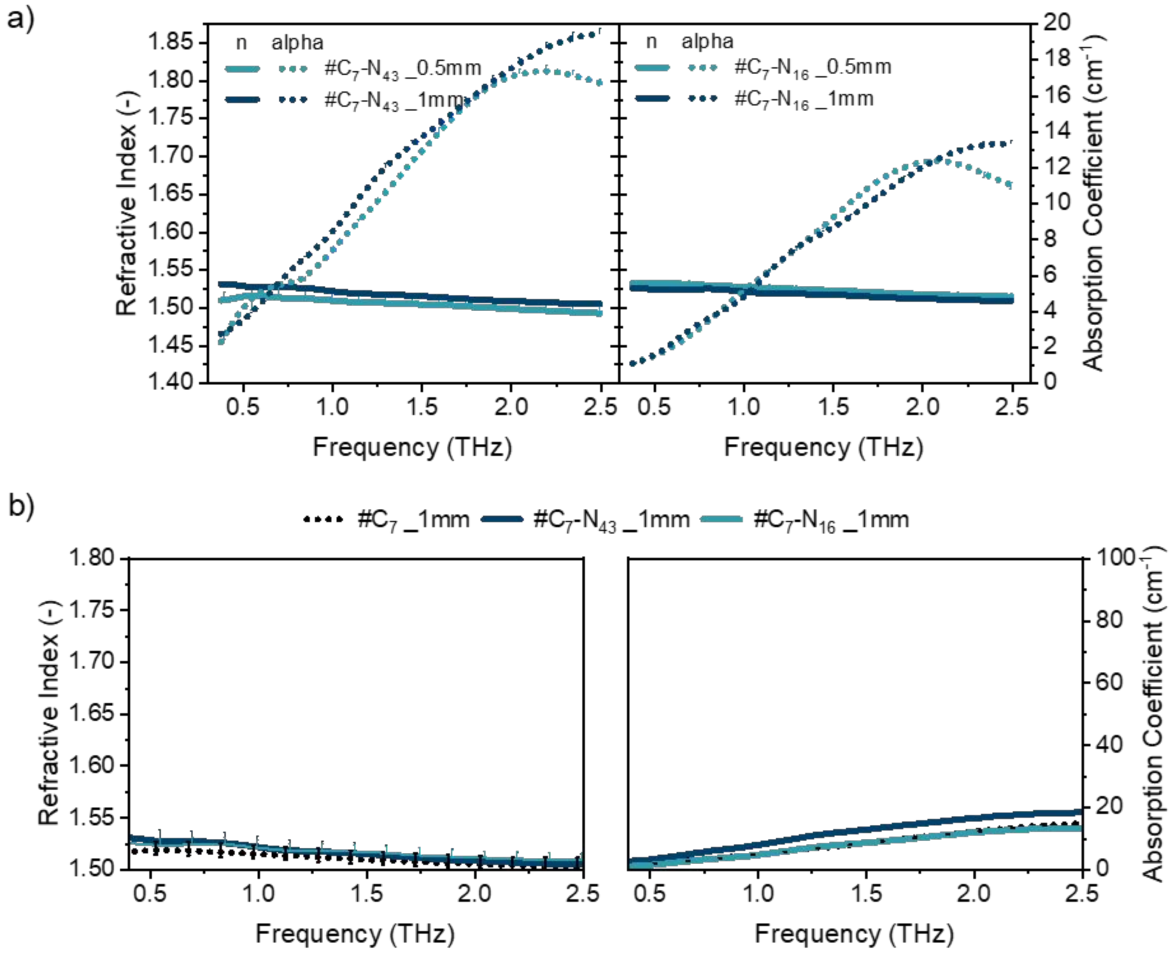

Figure S 5. Absorption coefficient and refractive index for #C_7-N_{43} and #C_7-N_{16}: (a) Spectra obtained from samples with varying thicknesses; (b) Comparative analysis of the optical parameters across different formulations, using samples 1mm-thick.

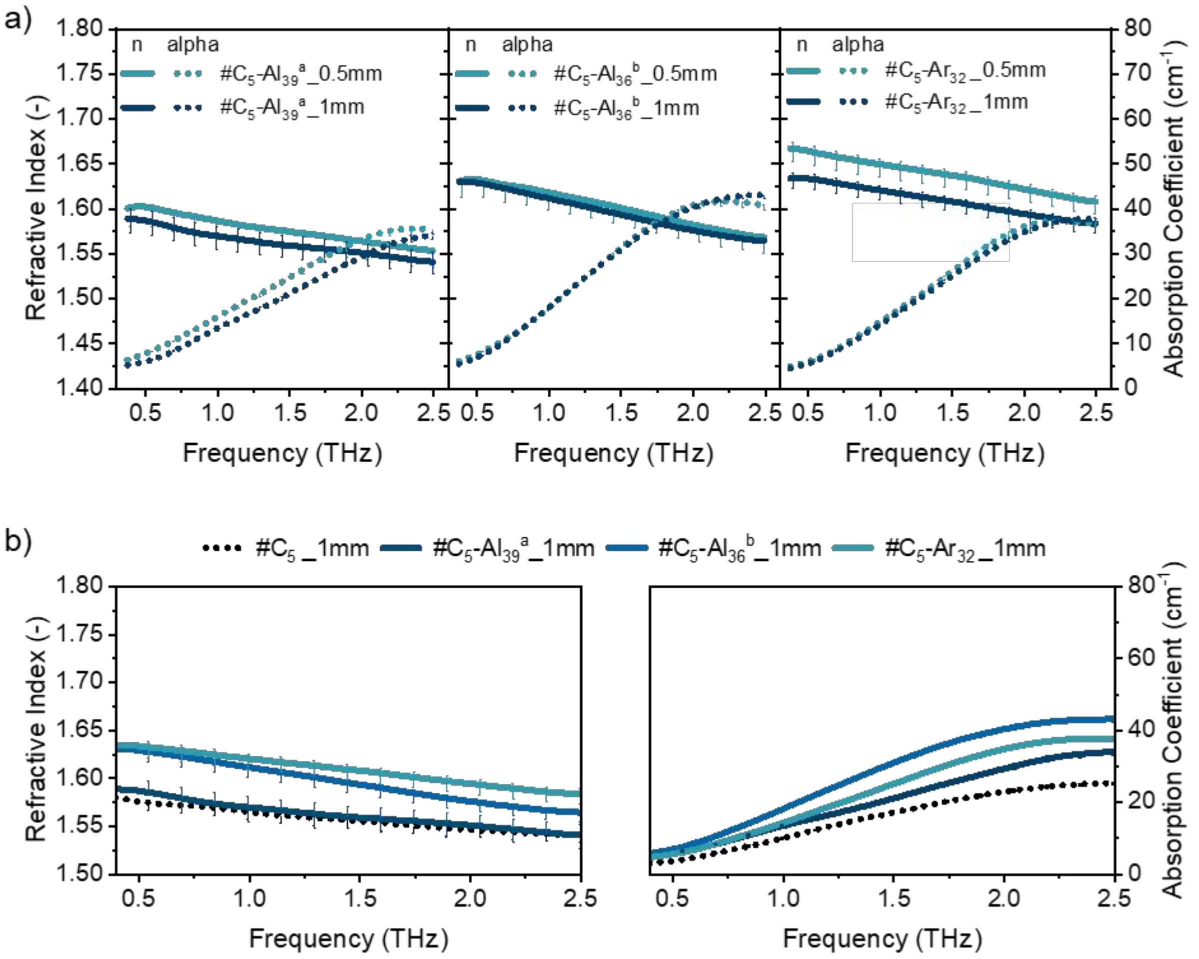

Figure S 6. Absorption coefficient and refractive index for formulations #C_5-Al_{39}^a to #C_5-Ar_{32}: (a) Spectra obtained from samples with varying thicknesses; (b) Comparative analysis of the optical parameters across different formulations, using samples 1mm-thick.

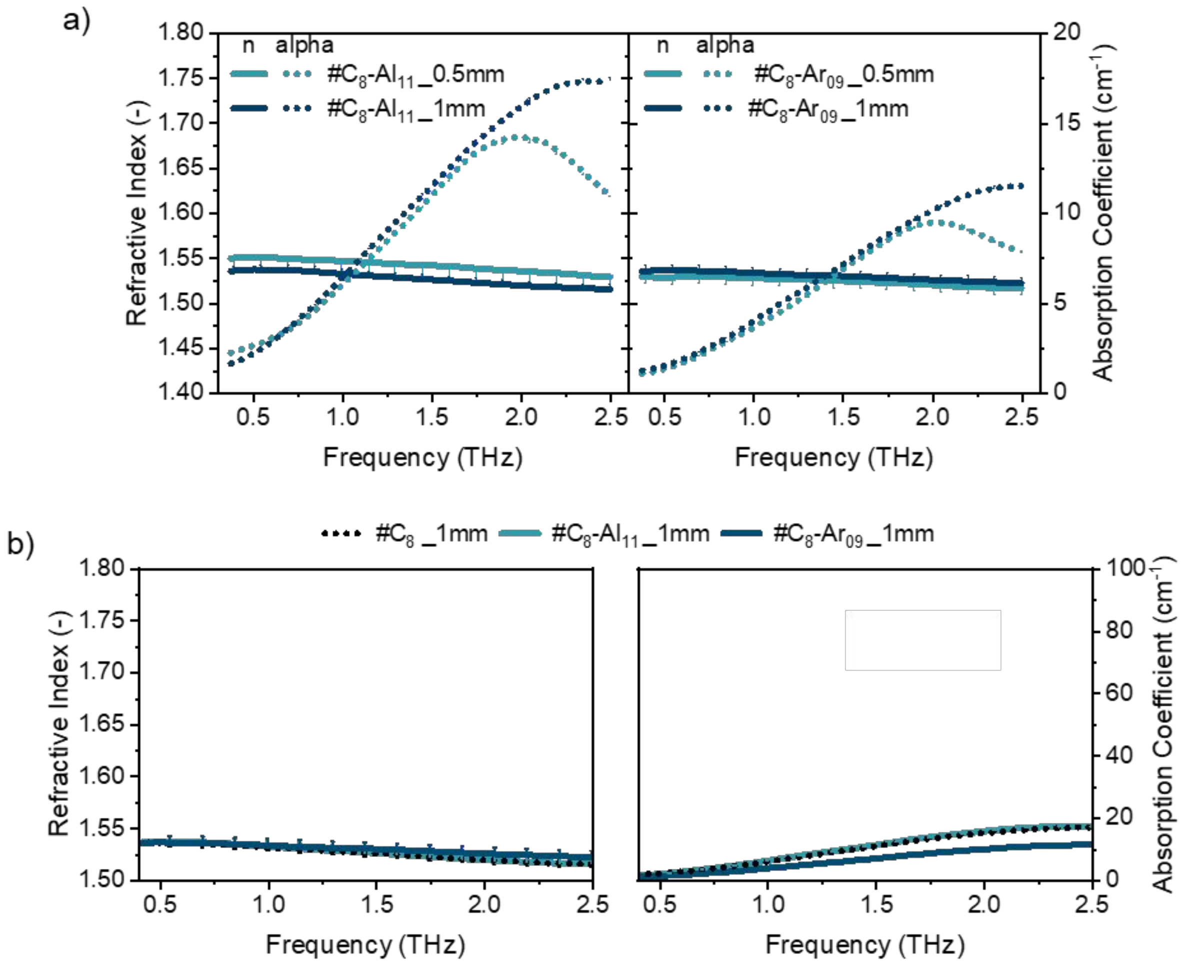

Figure S 7. Absorption coefficient and refractive index for formulations #C_8-Al_{11} and #C_8-Ar_{09}: (a) Spectra obtained from samples with varying thicknesses; (b) Comparative analysis of the optical parameters across different formulations, using samples 1mm-thick.

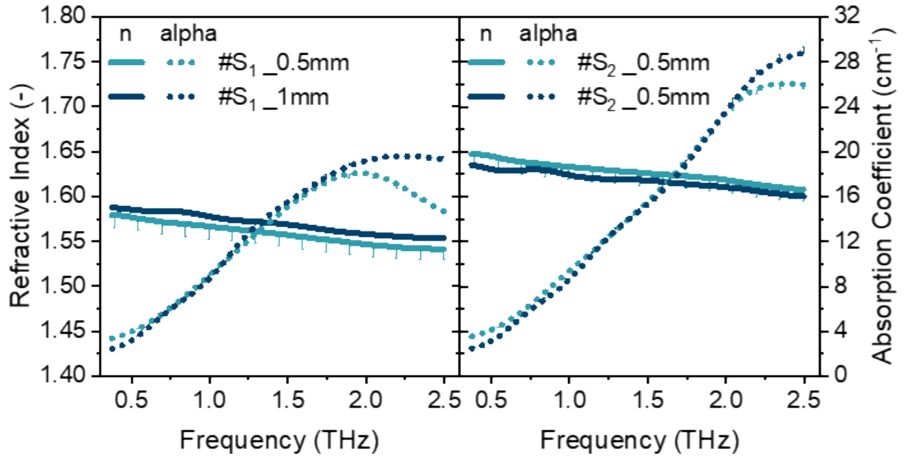

Figure S 8. Absorption coefficient and refractive index for #S_1 and #S_2, obtained from samples with varying thicknesses.

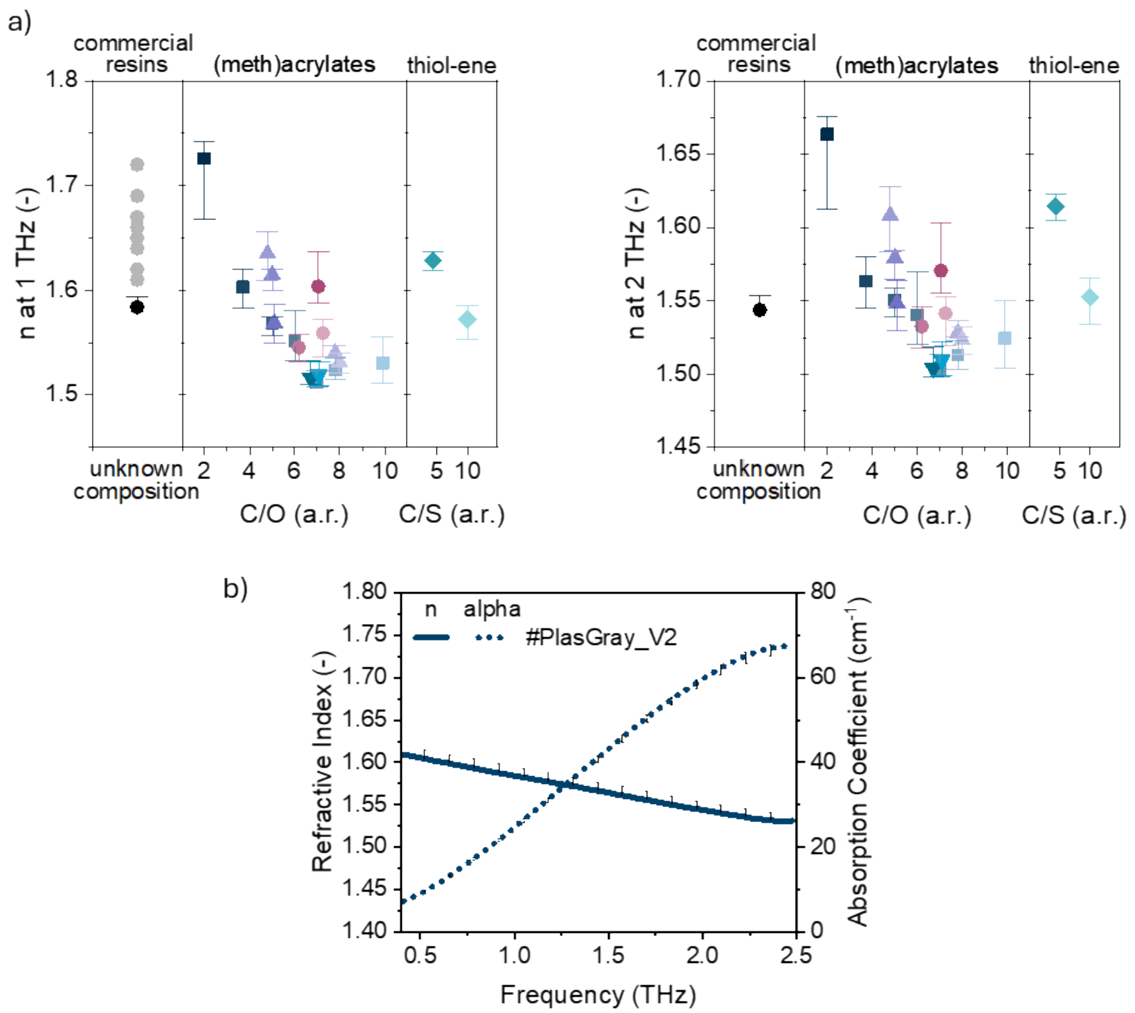

Figure S 9. a) Summary of the absorption coefficient at 1THz (left) and 2THz (right) of the studied polymers. Marker color and shape correspond to the formulations reported in Table 1. Commercial resins are included for comparison (black: #PlasGray_V2, gray: literature data from [4]
). b) Refractive index (n) and the Absorption coefficient (α) of 3D printed specimens obtained with formulations #PlasGray_V2.

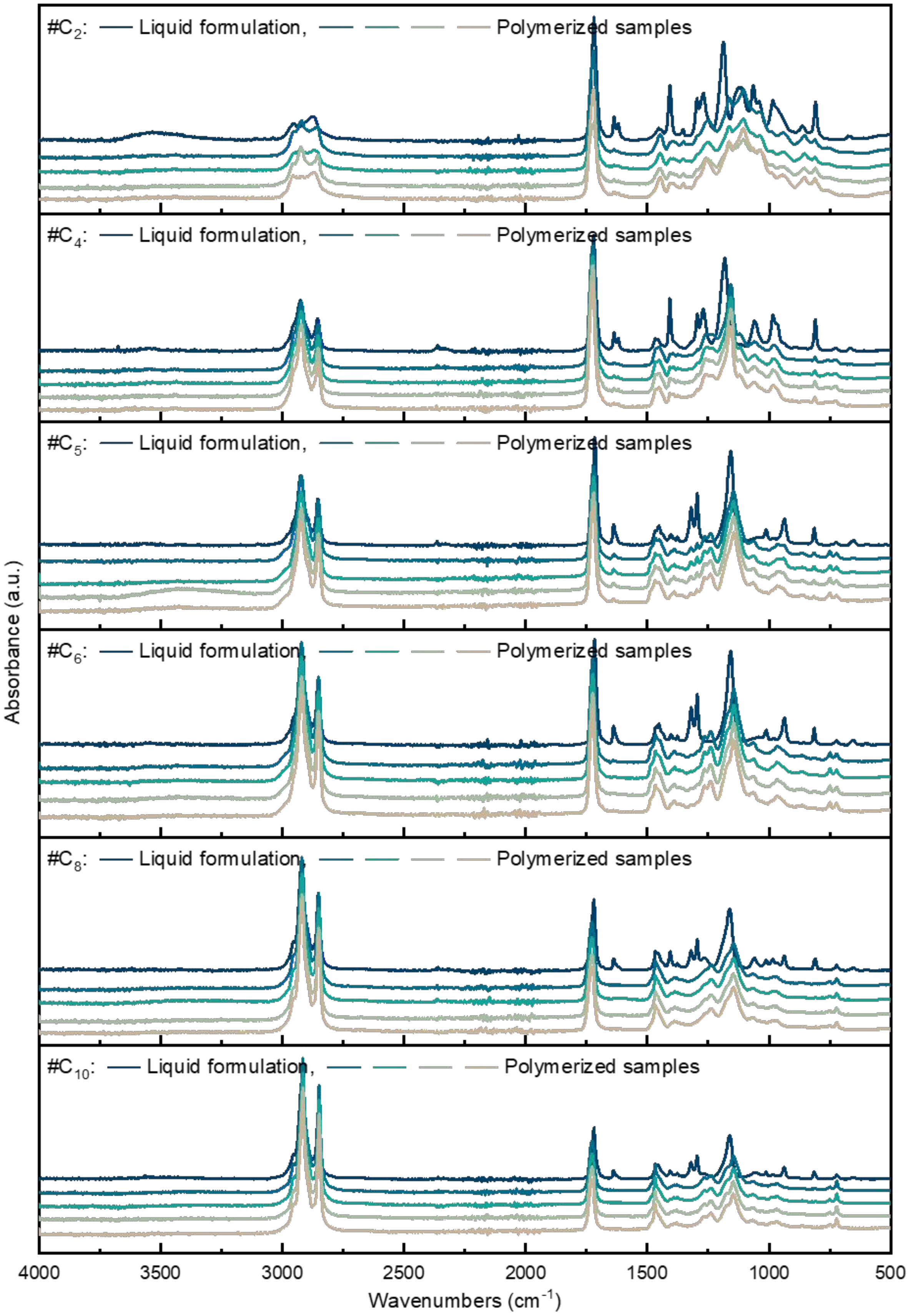

Figure S 10. ATR-FTIR spectra of formulations: #C_2, #C_4, #C_5, #C_6, #C_8, and #C_{10}. Different colors represent different samples.

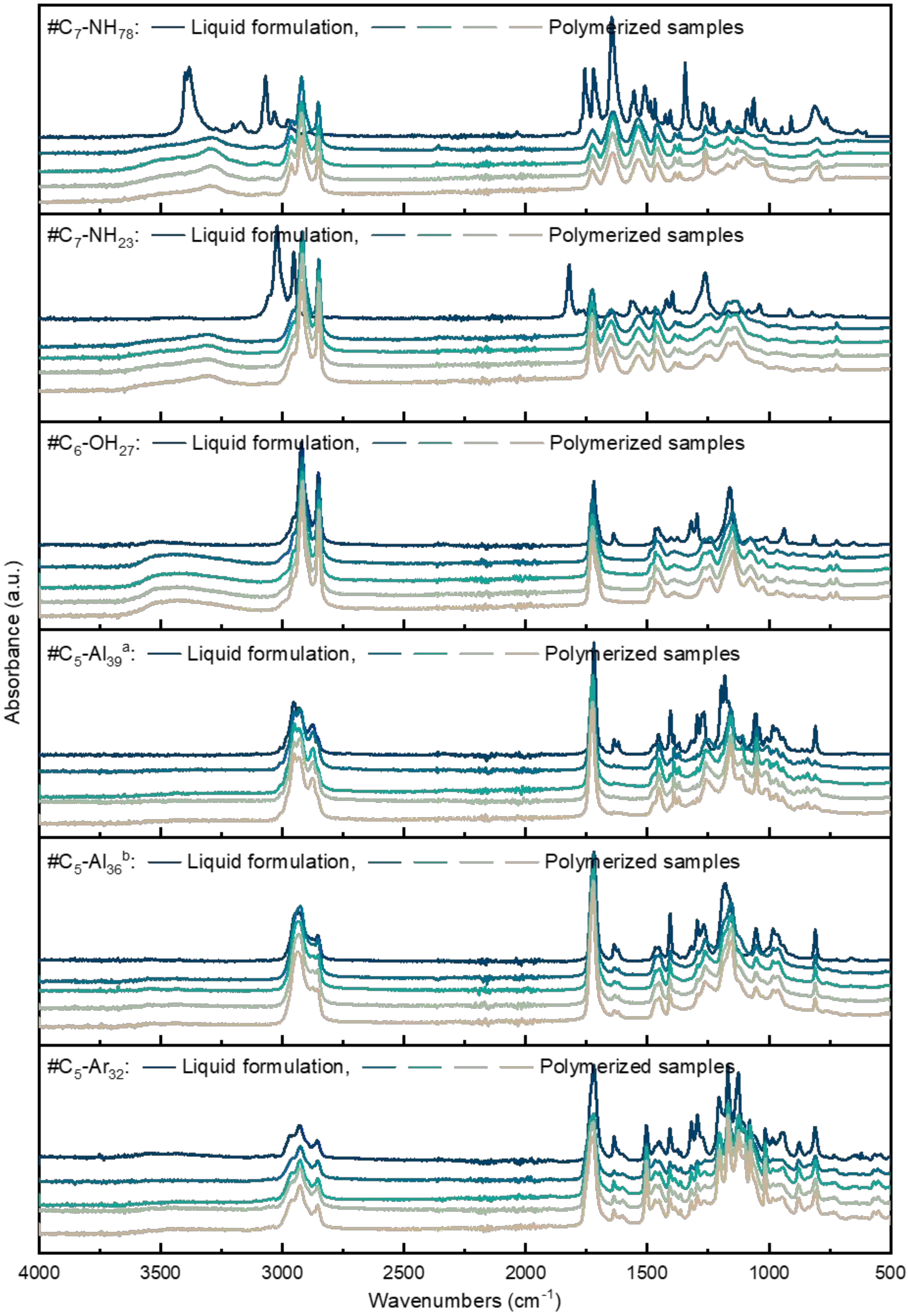

Figure S 11. ATR-FTIR spectra of formulations: #C_7-NH_{78}, #C_7-NH_{23}, #C_6-OH_{27}, #C_5-Al_{39}^a, #C_5-Al_{36}^b, and #C_5-Ar_{32}. Different colors represent different samples.

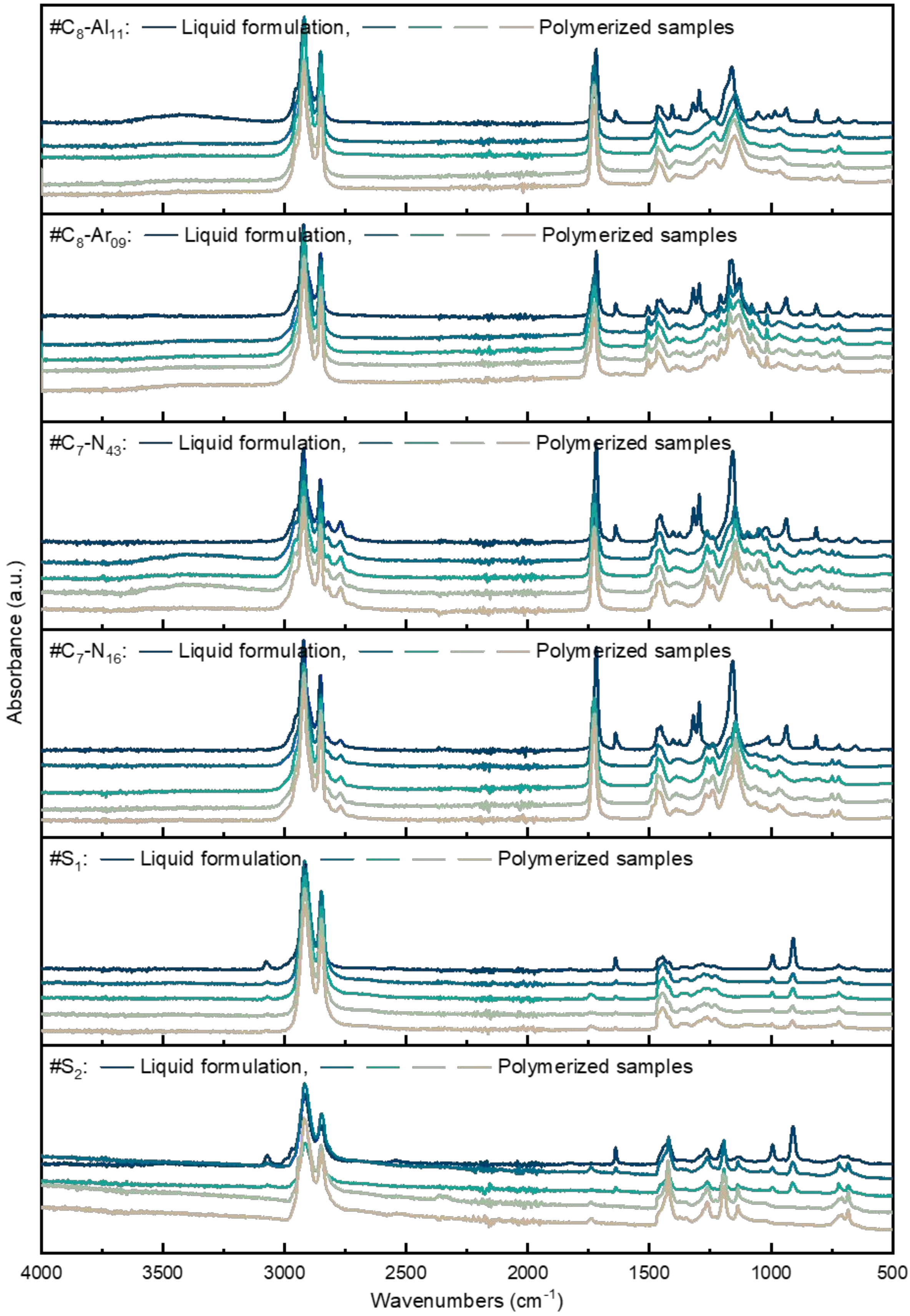

Figure S 12. ATR-FTIR spectra of formulations: #C_8-Al_{11}, #C_8-Ar_{09}, #C_7-N_{43}, #C_7-N_{16}, #S_1, and #S_2. Different colors represent different samples.

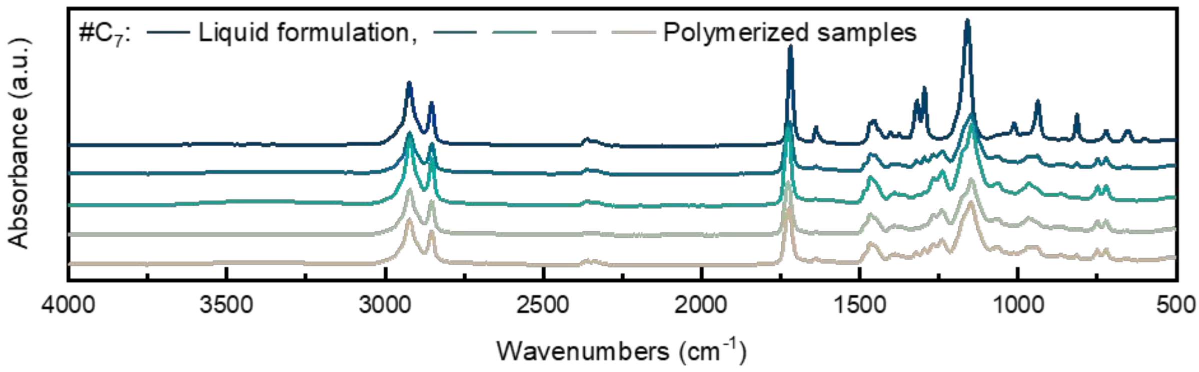

Figure S 13. ATR-FTIR spectra of formulation #C₇. Different colors represent different samples.

Formulation optimization and 3D printing

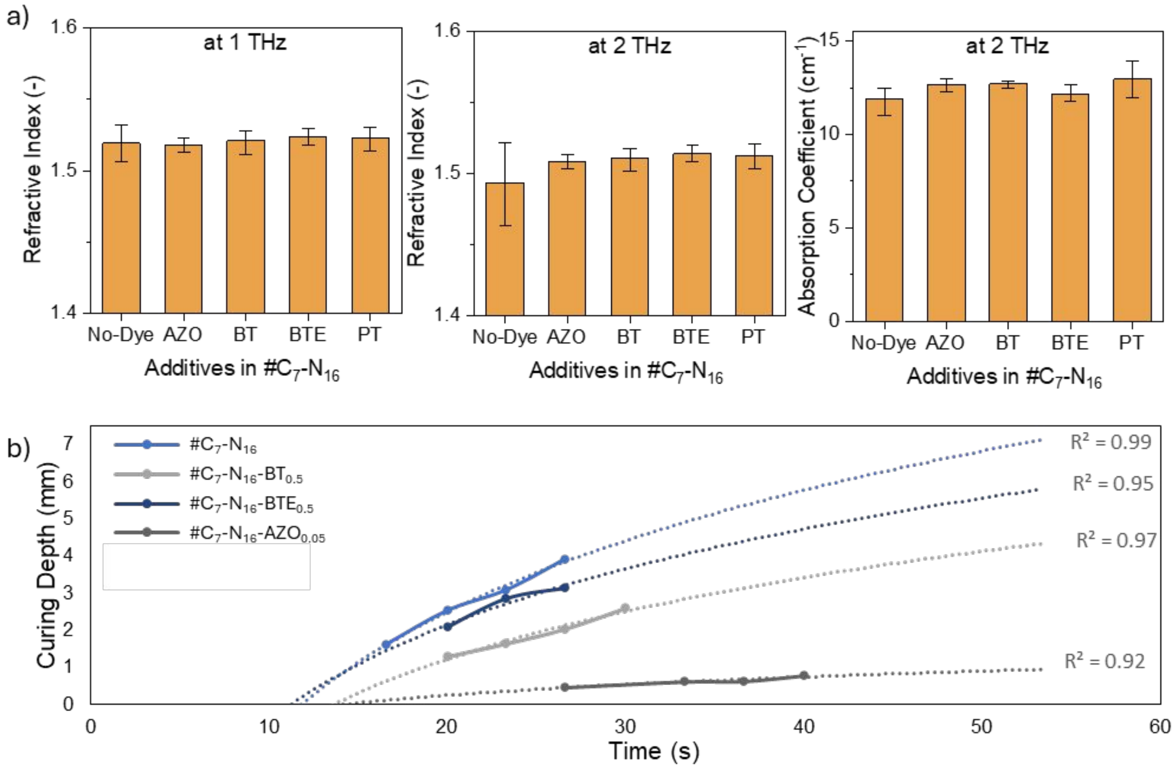

Figure S 14. Additives effect on #C_7-N_{16} THz optical parameters at 1 and 2 THz (a) and the resins working curves (b).

Table S 5. Chemical composition of the formulation optimized for the 3D printing.

Formulation name	Monomers (wt%)	Additive (phr)
#C_7-N_{16}-$AZO_{0.05}$-$PT_{0.1}$	DMAEMA 16%<br>DoDDMA 27%<br>ODMA 58%	0.05 AZO<br>0.1 PT
#C_7-$AZO_{0.05}$	DoDDMA 50%<br>ODMA 50%	0.05 AZO

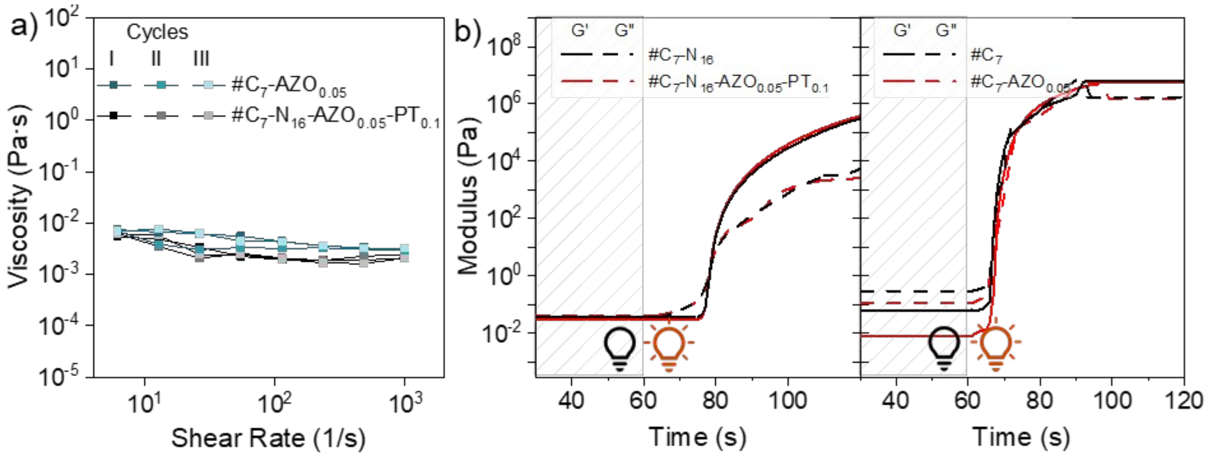

Figure S 15. a) Flow curves; b) Photorheology results.

Table S 6. 3D Printing parameters.

Formulation	Sample name	Range	Number of layers (-)	Layer thickness (μm)	Light Intensity (mW cm^{-2})	Irradiation time (s)
#C_7	Snowflake	Burn in	2	100	50	8
		Range 1	-	100	50	5.5
#C_7-N_{16}	Snowflake	Burn in	2	100	50	8
		Range 1	-	100	50	6
#C_7-$AZO_{0.5}$	Snowflake | Flat Sample	Burn in	2	50	50	6
		Range 1	-	50	50	10.5
	Cube	Burn in	4	30	50	5
		Range 1	-	30	50	10
	PhC_0.25	Burn in	4	30	50	5
		Range 1	-	30	50	11
	PhC_0.4	Burn in	4	30	50	5
		Range 1	-	30	50	9.8
#C_7-N_{16}-$AZO_{0.05}$-$PT_{0.1}$	Snowflake	Burn in	2	50	50	15.3
		Range 1	-	50	50	16
	PhC_0.25	Burn in	1	150	50	20
		Range 1	2	200	50	21.75
	Flat-25	Burn in	2	25	50	6
		Range 1	-	25	50	8.75
	Flat-50	Burn in	2	50	50	13
		Range 1	-	50	50	15.6
	Flat-100	Burn in	2	100	50	20
		Range 1	-	100	50	20.6
	Flat-150	Burn in	2	150	50	20
		Range 1	-	150	50	20
	Flat-200	Burn in	2	200	50	20
		Range 1	-	200	50	25
#PlasGray_V2	PhC_0.25	Burn in	1	50	60	4.062
		Range 1	-	50	60	0.725

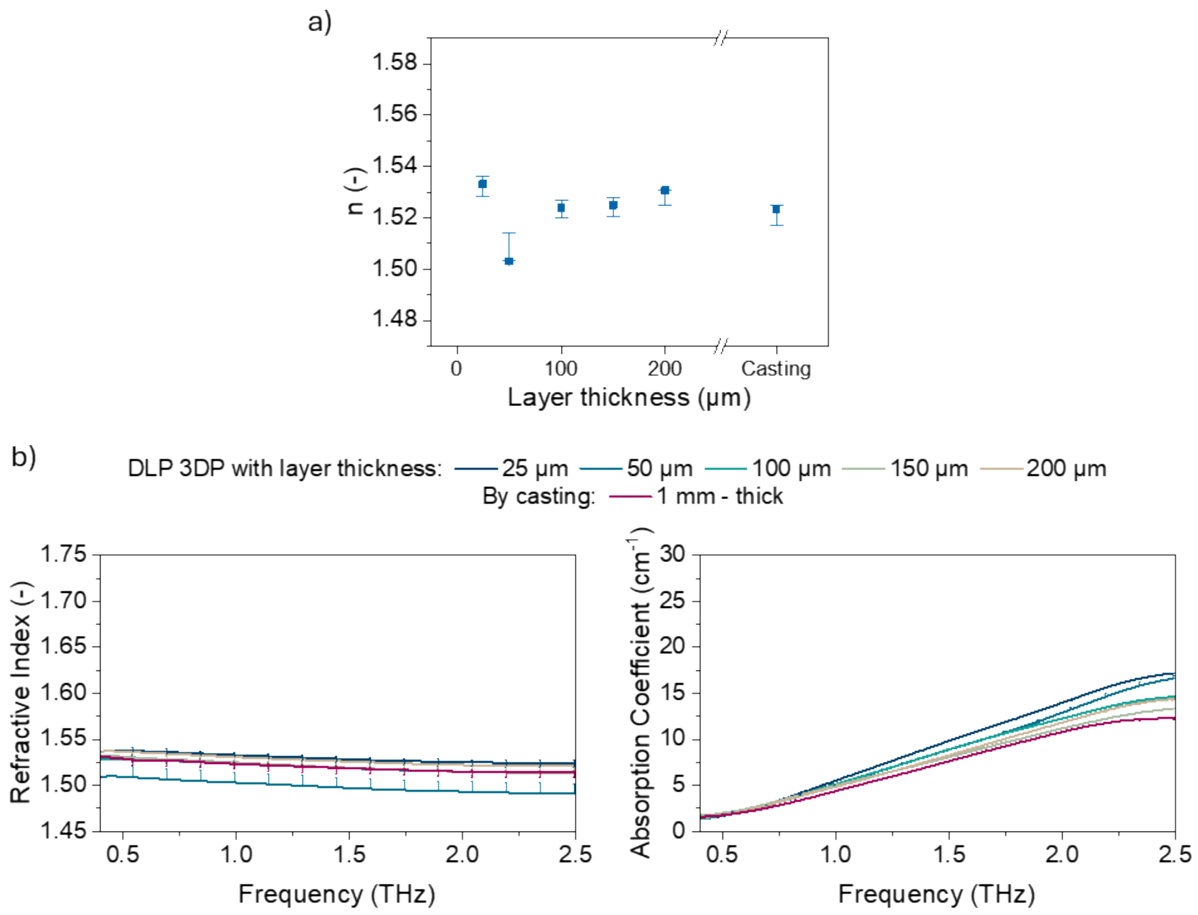

Figure S 16. a) Exponential decay fit of n at 1THz of #C_7-N_{16}-$AZO_{0.05}$-$PT_{0.1}$ samples obtained by 3D printing with different layer thickness compared to casting – all samples were 1mm thick; b) Comparative analysis of the optical parameters across #C_7-N_{16}-$AZO_{0.05}$-$PT_{0.1}$ samples 3D printed whit different layers thickness compared to a sample obtained by casting, all using 1mm-thick samples.

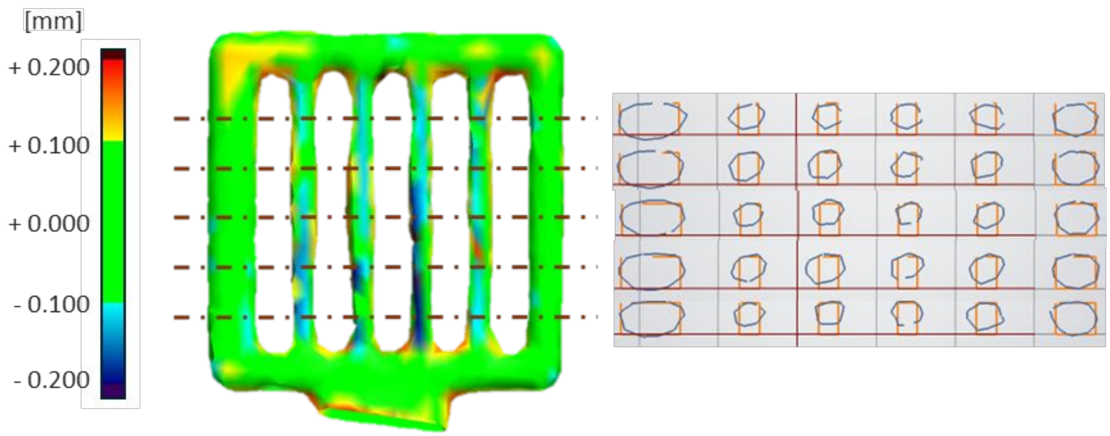

Figure S 17. #PlasGray_V2 structure: Printing fidelity heat map obtained comparing 3D-scanned model and the original CAD model, on the right the cross sections comparisons between the CAD model (orange) and the 3D-scanned model (blue) are reported.

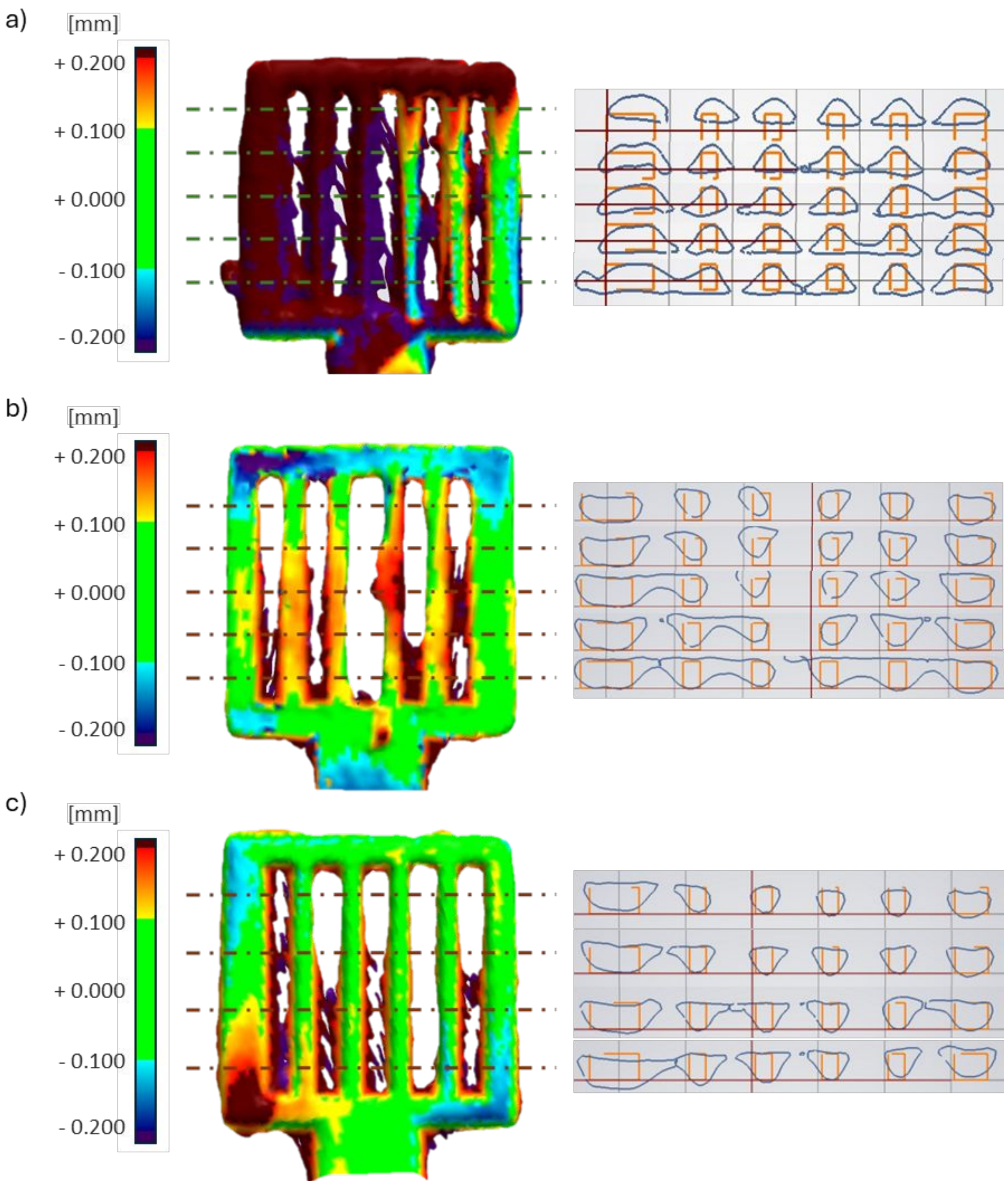

Figure S 18. Printing fidelity heat map obtained comparing 3D-scanned model and the original CAD model of #C_7-N_{16}-$AZO_{0.05}$-$PT_{0.1}$ PhC_0.25_#1 (a), PhC_0.25_#2 (b) and PhC_0.25_#3 (c) structures:, on the right the cross sections comparisons between the CAD model (orange) and the 3D-scanned model (blue) are reported.

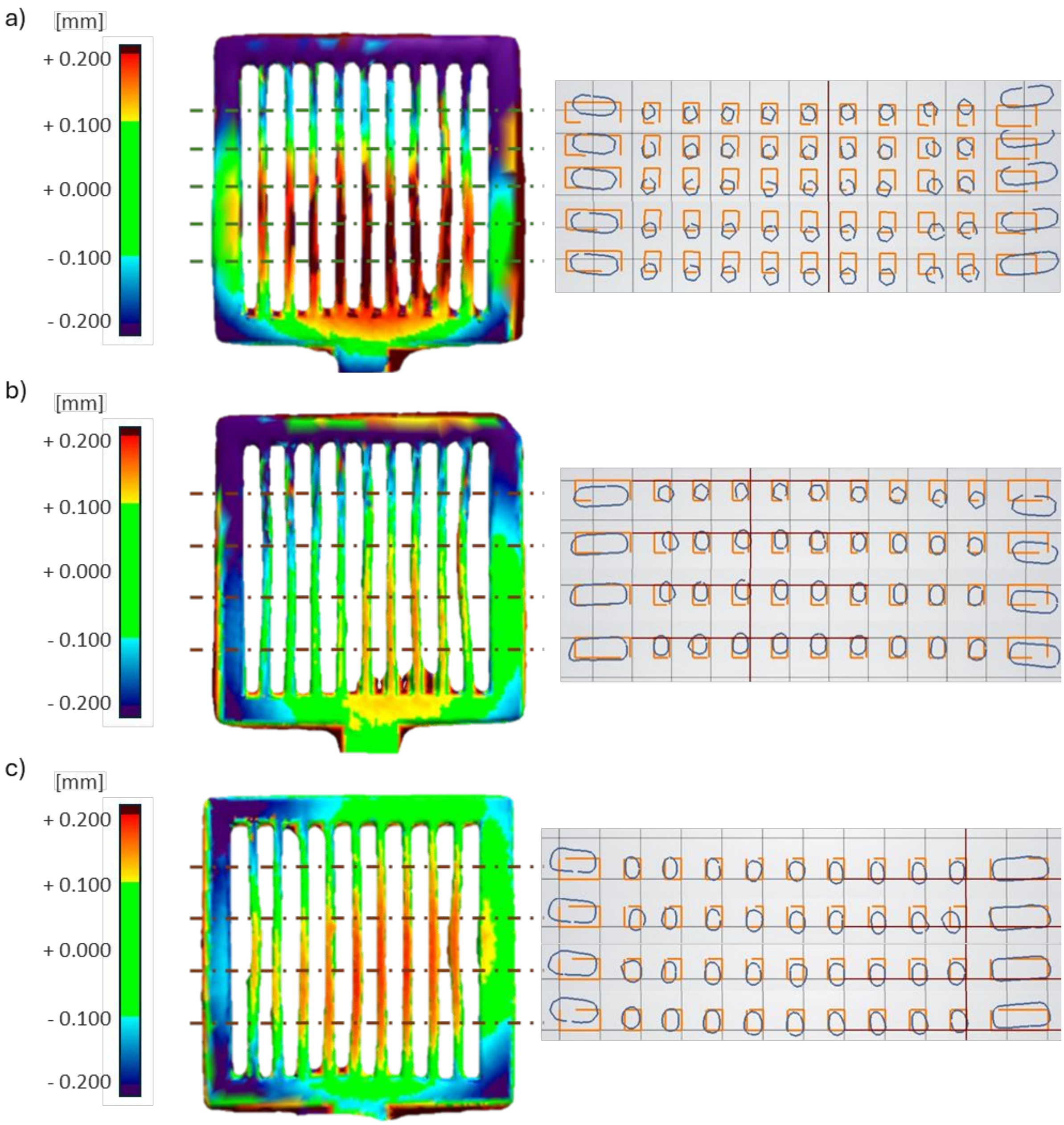

Figure S 19. Printing fidelity heat map obtained comparing 3D-scanned model and the original CAD model of #C_7-$AZO_{0.05}$ PhC_0.25_#1 (a), PhC_0.25_#2 (b) and PhC_0.25_#3 (c) structures, on the right the cross sections comparisons between the CAD model (orange) and the 3D-scanned model (blue) are reported.

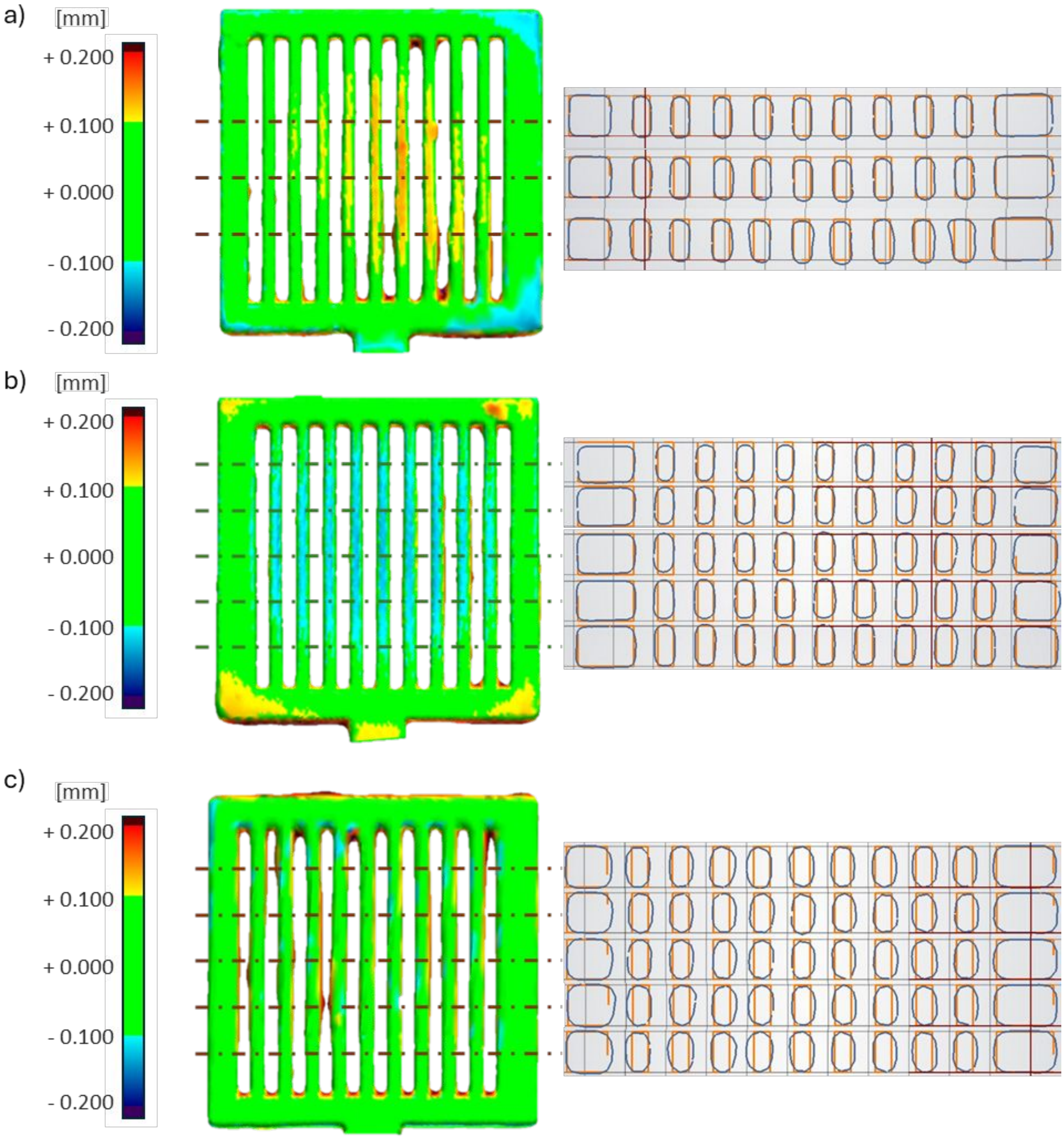

Figure S 20. Printing fidelity heat map obtained comparing 3D-scanned model and the original CAD model of #C_7-$AZO_{0.05}$ PhC_0.4_#1 (a), PhC_0.4_#2 (b) and PhC_0.4_#3 (c) structures:, on the right the cross sections comparisons between the CAD model (orange) and the 3D-scanned model (blue) are reported.

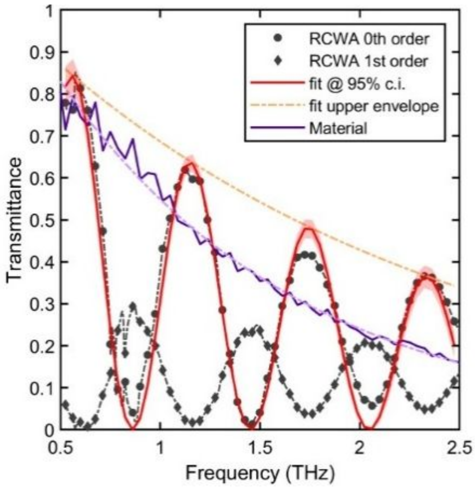

Figure S 21. Zeroth-order transmission spectrum at normal incidence, calculated by RCWA for polarization along the grating rods (black dots), together with a phenomenological fit (red) and its upper envelope (orange dashed-line). The first-order transmission spectrum calculated under the same conditions (black diamonds) and the material transmission (purple line) are also shown.

References

(1) Pupeza, I.; Wilk, R.; Koch, M. Highly Accurate Optical Material Parameter Determination with THz Time-Domain Spectroscopy. *Opt. Express* **2007**, *15* (7), 4335. https://doi.org/10.1364/OE.15.004335.

(2) Fastampa, R.; Pilozzi, L.; Missori, M. Cancellation of Fabry-Perot Interference Effects in Terahertz Time-Domain Spectroscopy of Optically Thin Samples. *Phys Rev A* **2017**, *95* (6), 063831. https://doi.org/10.1103/PhysRevA.95.063831.

(3) Lewis, R. A. Terahertz Imaging and Spectroscopy Methods and Instrumentation. In *Encyclopedia of Spectroscopy and Spectrometry*; Elsevier, 2017; pp 422–426. https://doi.org/10.1016/B978-0-12-409547-2.12146-8.

(4) Duangrit, N.; Hong, B.; Burnett, A. D.; Akkaraekthalin, P.; Robertson, I. D.; Somjit, N. Terahertz Dielectric Property Characterization of Photopolymers for Additive Manufacturing. *IEEE Access* **2019**, *7*, 12339–12347. https://doi.org/10.1109/ACCESS.2019.2893196.
